# Dietary and Formulation Potential of Natural Oils and Fats in Ocular Health and Disease: Mechanisms, Evidence, and Future Directions

**DOI:** 10.3390/nu18183007

**Published:** 2026-09-14

**Authors:** Jiexin Bai, Xiangyi Fan, Youruo Zhang, Junguo Duan

**Affiliations:** Eye Health with Traditional Chinese Medicine Key Laboratory of Sichuan Province, Retinal Image Technology and Chronic Vascular Disease Prevention & Control and Collaborative Innovation Center, Eye School of Chengdu University of TCM, Chengdu 610036, China; baijiexin@stu.cdutcm.edu.cn (J.B.); fanxiangyi@stu.cdutcm.edu.cn (X.F.); youruozhang@stu.cdutcm.edu.cn (Y.Z.)

**Keywords:** natural oils, ocular health, bioactive lipids, oxidative stress, ophthalmic formulations

## Abstract

Natural oils and fats are multicomponent lipid matrices composed predominantly of triacylglycerols with source-specific fatty-acid profiles, together with variable amounts of phospholipids and minor lipophilic constituents. This narrative review discusses the nutritional, biological, and pharmaceutical relevance of oils from plant, terrestrial animal, and marine sources in ocular health and disease. Whole natural oils and fats are distinguished from isolated oil-associated constituents, multi-ingredient formulations, pharmaceutical oil phases or excipients, and engineered lipid-delivery systems. The discussion focuses on fatty acids, phospholipids, carotenoids, tocopherols, phytosterols, phenolic compounds, and other lipophilic constituents in relation to tear-film stability, epithelial homeostasis, inflammatory regulation, retinal function, and ophthalmic delivery. The available literature encompasses human studies, experimental models, cell-based research, and formulation studies. Human research is concentrated mainly on ocular-surface outcomes and product-specific interventions, whereas the literature concerning most intraocular and retinal conditions is derived predominantly from preclinical or mechanistic studies. The review also considers the influence of oil source, composition, extraction, refining, cooking, oxidation, formulation characteristics, administration route, and safety on the interpretation of oil-related ocular research.

## 1. Introduction

Ocular health and good vision have wide-ranging and profound implications for daily life, health, employment, family functioning, society, and sustainable economic development [1]. According to the World Health Organization’s fact sheet updated in February 2026, at least 2.2 billion people worldwide have near or distance vision impairment. In at least 1 billion of these cases, accounting for almost half of the total, the impairment could have been prevented or has yet to be addressed [2]. The leading causes of blindness and vision impairment include age-related cataract; uncorrected refractive errors, particularly myopia and presbyopia; glaucoma; age-related macular degeneration (AMD); and diabetic retinopathy (DR) [3]. Among these conditions, cataract remains the leading cause of blindness globally across all 10-year age groups among people aged 50 years and older [4]. Myopia is one of the most common ocular disorders worldwide and typically develops during childhood and early adulthood [5,6]. Vision impairment caused by insufficient optical correction for presbyopia affects approximately 826 million people globally [7]. Glaucoma is a progressive neurodegenerative disease and the most common cause of irreversible blindness [8]. AMD is a major cause of severe vision impairment in older adults and is projected to affect approximately 288 million people worldwide by 2040 [9,10,11]. DR is a common complication of diabetes and a leading cause of preventable blindness among working-age adults [12,13,14]. Acute and chronic ocular surface diseases, particularly dry eye disease (DED), are directly shaped by—and interact with—personal lifestyle, working environments, mental health, access to medical resources, economic status, and educational level in the information technology era [15,16,17,18]. Although the global age-standardised prevalence of blindness has declined, the crude prevalence is increasing. With population growth, ageing, and the expansion of digital lifestyles, ocular health remains an urgent and increasingly important public-health priority.

The onset and progression of ocular diseases are rarely driven by a single factor. Instead, they are closely linked to shared pathological processes, including oxidative stress, inflammation, mitochondrial dysfunction, lipid metabolic dysregulation, epithelial barrier disruption, and neurodegenerative injury [19,20]. These mechanisms contribute to tissue damage in diseases of the ocular surface, lens, retina, and optic nerve, and they may also provide biologically plausible entry points for research into nutritional interventions, natural bioactive compounds, and lipid-based formulations. In recent years, growing attention has been directed towards strategies that use dietary patterns and natural products to prevent, delay, or support the management of chronic diseases [21,22,23,24]. As important lipid resources in human diets and the food industry, natural oils and fats are not only sources of energy and essential fatty acids but may also provide a material basis linking nutritional modulation, functional-food development, bioactive-lipid research, and the design of ophthalmic formulations [25].

Compared with single nutrients, natural oils and fats are characterised by diverse origins, complex composition, variable bioavailability, and potential suitability for diverse formulation applications. Their composition includes major lipid classes, such as triacylglycerols (TAGs) and phospholipids with characteristic fatty-acid profiles, along with variable amounts of tocopherols, tocotrienols, carotenoids, phytosterols, squalene, phenolic compounds, and other minor lipophilic constituents. These components may influence ocular homeostasis through modulation of redox balance, inflammatory mediator metabolism, cell–membrane architecture, the tear-film lipid layer, mitochondrial function, and the delivery of lipophilic bioactive substances. At the same time, the composition and function of natural oils and fats are jointly shaped by species origin, extraction method, degree of refining, oxidation status, storage conditions, and route of exposure or administration. Their ocular relevance should therefore not be inferred from source category or dominant fatty-acid profile alone, but it should be evaluated through an integrated framework that includes compositional characterization, quality control, bioavailability, and application form [26].

Despite growing interest, evidence concerning natural oils and fats in ocular health remains fragmented and difficult to interpret. Oils from different sources vary substantially in lipid composition, minor bioactive constituents, processing history, oxidative stability, bioavailability, and formulation compatibility and should therefore not be regarded as interchangeable. More importantly, the existing literature encompasses fundamentally different interventions, including whole natural oils and fats, isolated oil-associated constituents, multi-ingredient nutritional or ophthalmic formulations, pharmaceutical oil phases or excipients, and engineered lipid-delivery systems. Relevant studies span dietary intake, oral supplementation, topical ocular application, and pharmaceutical formulation, and they include human clinical and observational studies, animal models, cell-based experiments, and formulation research. Considerable heterogeneity exists in the characterisation of the tested materials, dose, duration, comparator, study design, and disease-specific endpoints. In this review, whole natural oils and fats are treated as the central biological exposure, whereas evidence concerning isolated constituents, combined formulations, pharmaceutical excipients, and lipid-delivery systems is classified and discussed separately. Findings from one intervention category are not automatically attributed to another. Accordingly, this narrative review brings together and organises the available literature on the composition, quality attributes, ocularly relevant mechanisms, direct ocular evidence, routes of application, safety considerations, and translational limitations of plant-, terrestrial animal-, and marine-derived natural oils and fats.

## 2. Review Scope, Literature Search, and Evidence Classification

### 2.1. Scope Definition and Intervention Classification

This review examines two related but conceptually distinct domains: the biological and nutritional effects of natural oils and fats, and their pharmaceutical roles in ophthalmic formulations. For the purposes of this review, whole natural oils and fats were defined as recognisable multicomponent lipid matrices obtained from plant, terrestrial animal, marine, or microbial sources and evaluated as complete oils or fats rather than as purified individual compounds. Processing by mechanical pressing, aqueous or enzymatic extraction, solvent extraction, supercritical-fluid extraction, or conventional refining did not exclude an intervention from this category, provided that it remained a multicomponent oil matrix.

To prevent evidence obtained with fundamentally different materials from being conflated, all included studies were classified according to the material actually tested: (1) whole natural oils or fats; (2) isolated or defined oil-associated constituents and chemically characterised bioactive extracts, including individual fatty acids, carotenoids, phenolic compounds, vitamins, phospholipids, and defined constituent combinations; (3) multi-ingredient nutritional or ophthalmic formulations; (4) natural oils or derived lipids used primarily as pharmaceutical oil phases or excipients; and (5) engineered lipid-delivery systems, including nanoemulsions, liposomes, solid lipid nanoparticles, and nanostructured lipid carriers. Concentrated or purified EPA/DHA preparations were classified as constituent-level interventions rather than automatically as whole fish–oil interventions.

Evidence was interpreted within, rather than across, these categories. Findings obtained with an isolated constituent were not attributed directly to the whole oil in which that constituent may occur. Effects observed with multi-ingredient products were interpreted at the formulation level unless the independent contribution of an individual component had been demonstrated. Similarly, improvements in drug solubility, stability, ocular retention, permeability, or controlled release were considered evidence of pharmaceutical or delivery performance and not evidence of an inherent therapeutic effect of the carrier oil.

### 2.2. Literature Search and Evidence Selection

This narrative review was supported by a comprehensive literature search of PubMed/MEDLINE, Web of Science Core Collection, Scopus, and Embase from database inception to August 2026. The search was intended to support broad and transparent identification of the relevant literature rather than to meet the methodological requirements of a systematic review.

The search strategy combined terms relating to natural oils and fats, oil-associated constituents, ocular tissues and diseases, and ophthalmic formulations. Oil-related terms included “natural oil”, “edible oil”, “plant oil”, “seed oil”, “fruit oil”, “animal fat”, “marine oil”, “fish oil”, “algal oil”, “krill oil”, “cod liver oil”, “olive oil”, “flaxseed oil”, “castor oil”, “sesame oil”, “soybean oil”, “coconut oil”, “palm oil”, “sea buckthorn oil”, and “egg yolk oil”. Constituent-related terms included “fatty acid”, “omega-3”, “omega-6”, “eicosapentaenoic acid”, “docosahexaenoic acid”, “α-linolenic acid”, “linoleic acid”, “γ-linolenic acid”, “phospholipid”, “carotenoid”, “tocopherol”, “tocotrienol”, “phytosterol”, “squalene”, “polyphenol”, “hydroxytyrosol”, and “sesamol”. These terms were combined with ocular terms, including “eye”, “ocular”, “ophthalmic”, “ocular surface”, “tear film”, “dry eye”, “meibomian gland dysfunction”, “cornea”, “conjunctiva”, “uveitis”, “cataract”, “retina”, “age-related macular degeneration”, “diabetic retinopathy”, “glaucoma”, “retinitis pigmentosa”, “retinal degeneration”, and “optic nerve”. Additional targeted searches incorporated formulation- and quality-related terms, including “topical”, “eye drop”, “emulsion”, “nanoemulsion”, “liposome”, “solid lipid nanoparticle”, “nanostructured lipid carrier”, “lipid nanoparticle”, “nanomicelle”, “drug delivery”, “extraction”, “refining”, “processing”, “oxidation”, “stability”, and “safety”. The terms were combined using Boolean operators, and the syntax was adapted to the requirements of each database. Reference lists of relevant reviews, meta-analyses, and eligible primary studies were also examined to identify additional publications. No restriction by publication year was applied, although recent studies were prioritised when they provided evidence of comparable or greater direct relevance.

Eligible publications included human clinical trials, observational studies, ocular animal models, ocular cell-based studies, and formulation studies directly related to ocular health, ocular disease, ophthalmic delivery, or the safety and quality of oil- or lipid-containing interventions. Reviews and meta-analyses were used to provide background, identify relevant primary studies, and contextualise areas in which the findings were extensive or inconsistent. General food-processing and industrial studies were retained only when they provided information directly relevant to oil composition, oxidative status, bioavailability, safety, or ophthalmic formulation performance. Non-ocular mechanistic studies were included selectively when direct ocular evidence was limited or unavailable and were identified as indirect evidence. Studies without a clear relationship to ocular outcomes or ophthalmic formulations, purely industrial applications without ocular relevance, conference abstracts lacking sufficient methodological information, and duplicate publications were excluded. Evidence derived from whole foods or broad dietary patterns was not treated as direct evidence for the efficacy of a specific oil unless the contribution of that oil could be distinguished from that of the overall dietary exposure.

After duplicate removal, titles and abstracts were screened for relevance, followed by full-text examination of potentially eligible publications. Study selection considered relevance to the review scope, identification of the material actually tested, directness to ocular outcomes, study design, and availability of sufficient information on the intervention and outcomes. For human studies, information on the population, study design, sample size, comparator, dose, duration, ocular endpoints, and principal findings was recorded. For animal- and cell-based studies, the model, intervention, dose or concentration, duration, and principal ocular outcomes were considered. Formulation studies were examined according to the active agent, function of the oil or lipid, delivery system, formulation performance, and reported ocular safety or tolerability.

## 3. Natural Oils and Fats as Multicomponent Lipid Matrices: Composition, Quality, and Ocular Relevance

### 3.1. Composition and Physicochemical Characteristics of Natural Oils and Fats

The natural oils and fats considered in this Review are derived primarily from plant seeds and fruits, terrestrial animal tissues and secretions, and marine organisms, including microalgae [27,28,29]. Most edible oils and fats are dominated by TAGs, in which fatty acids are esterified to a glycerol backbone. They also contain varying amounts of free fatty acids, phospholipids, sphingolipids, sterols, wax esters, tocopherols and tocotrienols, carotenoids, squalene, phenolic lipids, fat-soluble vitamins, and other minor constituents [30,31,32]. The relative abundance of these components differs substantially among oil sources. Although most conventional edible oils are TAG-rich, egg-derived lipid fractions may contain higher proportions of phospholipids, whereas lanolin is composed predominantly of wax esters, sterol esters, and related non-TAG lipids.

The physicochemical properties of an oil depend not only on its total fatty-acid composition but also on fatty-acid chain length, degree and position of unsaturation, cis–trans configuration, TAG molecular species, and the positional distribution of fatty acids on the glycerol backbone. These structural characteristics influence melting and crystallisation behaviour, fluidity, viscosity, interfacial properties, digestibility, and susceptibility to oxidation. Oils enriched in long-chain saturated fatty acids generally have higher melting points and greater solid-fat contents, whereas increasing unsaturation usually enhances fluidity but also increases oxidative susceptibility. Polar lipids, waxes, and sterols can further influence interfacial organisation, emulsification, spreading, and phase behaviour, while pigments and other minor constituents contribute to colour, flavour, antioxidant capacity, and storage stability [33,34]. Lipidomic approaches therefore provide more detailed compositional information than conventional measurements of total fat or broad fatty-acid classes by resolving individual lipid species, acyl-chain architecture, and polar-lipid fractions.

Oil composition is influenced by species, cultivar or breed, anatomical tissue, maturity, geographical and environmental conditions, and seasonal variation [35,36,37]. Extraction, refining, heating, storage, and interactions with the surrounding food matrix can further alter the proportions and availability of major and minor constituents [38,39,40].

In addition to compositional analysis, evaluation of oil quality commonly includes acid value, peroxide value, p-anisidine value, total oxidation value, oxidative induction period, moisture and volatile matter, and the presence of contaminants or processing-derived compounds. Reporting these characteristics is particularly important when comparing oils across nutritional, experimental, and formulation studies because products identified by the same source name may differ considerably in fatty-acid profile, minor constituents, oxidation status, and physical behaviour.

### 3.2. Mechanistic Basis Linking Natural Oils to Ocular Diseases

The ocular relevance of natural oils is best considered in the context of the tissue-specific functions of lipids in the eye. Lipids contribute to tear-film organisation, cellular and organelle membranes, lens membrane stability, photoreceptor outer-segment renewal, retinal energy metabolism, the visual cycle, and the generation of locally active lipid mediators [41,42,43]. However, the ocular surface, lens, retina, and retinal pigment epithelium (RPE) differ substantially in lipid composition, turnover, exposure to oxygen and light, and capacity for repair. Consequently, changes in lipid availability, organisation, and oxidation may have different implications in different ocular compartments.

**Figure 1:**
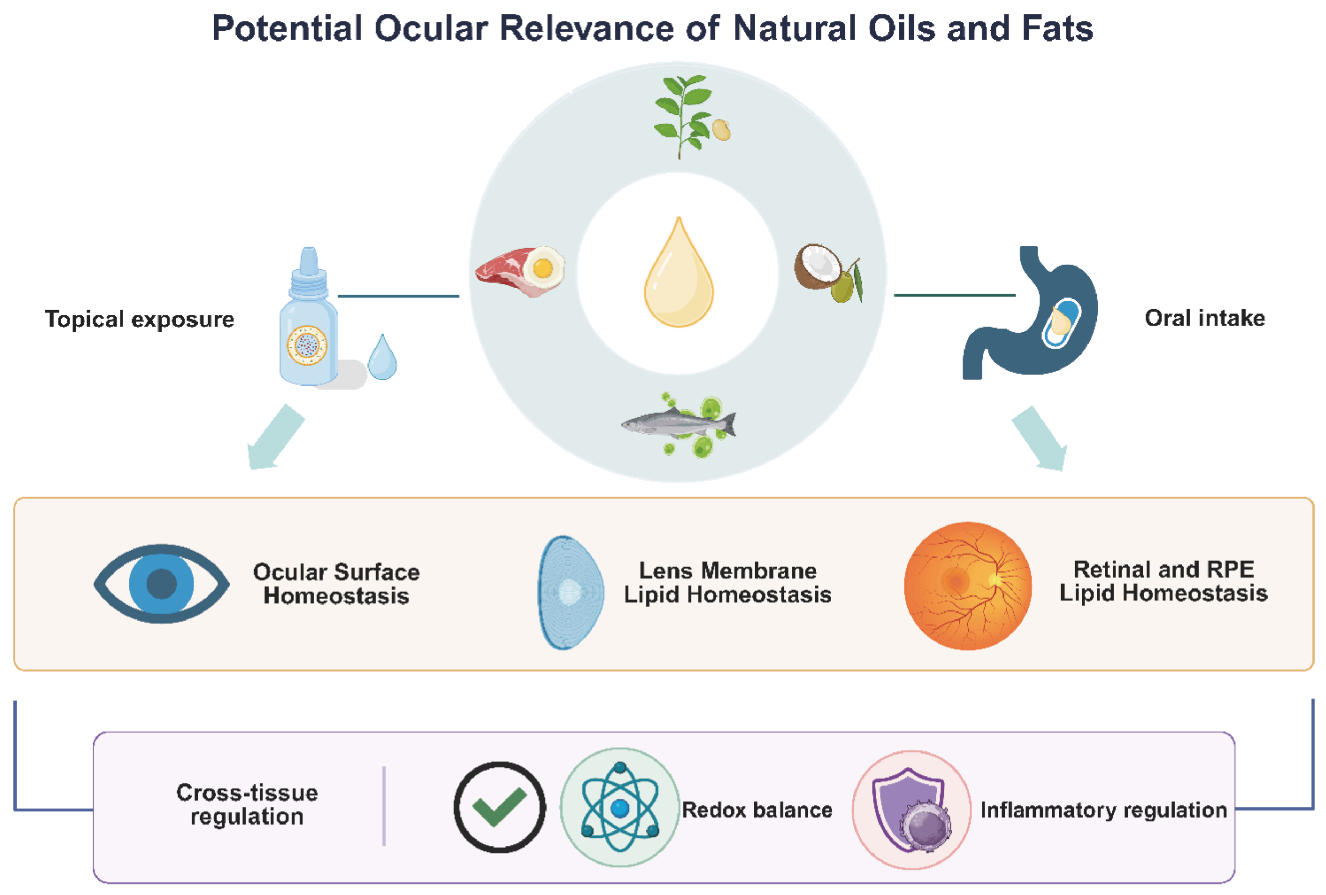
Tissue-based framework for the potential ocular relevance of natural oils and fats. Natural oils and fats derived from plant, terrestrial animal, and marine sources may influence ocular physiology through systemic lipid and micronutrient availability following oral intake or through direct interaction with the ocular surface when selected oils or oil-containing products are applied topically. Their potential relevance is organised across three ocular compartments: ocular-surface homeostasis, lens-membrane lipid homeostasis, and retinal and retinal pigment epithelium lipid homeostasis. Redox balance and inflammatory regulation represent shared processes across these ocular tissues. The relationships shown may vary according to oil source, composition, processing quality, oxidative stability, dose, and route of exposure. Created in BioRender. Bai, J. (2026) https://BioRender.com/imvgpq7.

Natural oils and fats provide source-dependent combinations of fatty acids and variable amounts of phospholipids, tocopherols, carotenoids, sterols, phenolic compounds, and other lipophilic constituents [31,32]. Following oral intake, these materials may influence systemic fatty-acid and lipid-soluble micronutrient availability, whereas topical oil-containing products can interact more directly with the tear film and ocular surface. Their ocular relevance may therefore be considered along an anatomical sequence extending from the ocular surface to the lens and posterior segment, together with shared redox and inflammatory processes that operate across these tissues (Figure 1).

#### 3.2.1. Tear-Film Lipid Layer and Ocular-Surface Homeostasis

The tear-film lipid layer represents the most direct connection between lipids and anterior ocular–surface function. Lipids secreted predominantly by the meibomian glands spread over the aqueous tear phase during blinking, contribute to surface tension and optical smoothness, and restrict excessive evaporation. Alterations in the quantity, composition, phase behaviour, or spreading of meibomian lipids can destabilise the tear film and are closely associated with evaporative DED and meibomian gland dysfunction (MGD) [44,45,46]. Increased evaporation may subsequently promote tear hyperosmolarity, epithelial stress, inflammatory signalling, ocular discomfort, and surface staining [47,48]. Oil-containing eye drops and ophthalmic emulsions are intended to supplement or stabilise this lipid-dependent interface. Depending on their composition and physical properties, the oil phase may enhance lubrication and spreading, reduce evaporative water loss, and prolong residence on the ocular surface [49,50,51]. Dietary lipids may influence the ocular surface through a less direct route. Fatty acids absorbed from the diet contribute to circulating lipid pools and may affect the substrates available for meibomian–lipid synthesis and lipid–mediator formation. Long-chain omega-3 fatty acids and their downstream mediators have also been studied in relation to corneal-nerve integrity, epithelial repair, tear secretion, and resolution of ocular–surface inflammation [52]. Thus, the potential ocular–surface relevance of oils involves both direct physicochemical support of the tear-film lipid layer and nutritional modulation of lipid-dependent epithelial, neural, and inflammatory processes.

#### 3.2.2. Lens Membrane Lipid Homeostasis

Maintenance of lens transparency depends on the long-term stability of crystallin proteins and the specialised membranes of lens fibre cells. These membranes have a distinctive lipid composition and organisation that contribute to permeability, protein–lipid interactions, ionic homeostasis, and optical properties. Because mature lens fibre cells have limited capacity for renewal, oxidative modification of membrane lipids and crystallins may accumulate during ageing [53,54,55,56].

Lipid peroxidation, glutathione depletion, protein oxidation, crystallin aggregation, and altered membrane organisation can disturb lens homeostasis and contribute to progressive loss of transparency. Changes in lens epithelial cell function and cellular senescence may further promote inflammatory signalling, extracellular matrix remodelling, and posterior capsular opacification [57,58,59].

The potential nutritional relevance of natural oils to the lens arises primarily from their contribution to systemic lipid and antioxidant status. Oils that retain tocopherols, carotenoids, phenolic compounds, and other antioxidant-active constituents may increase the dietary availability of compounds involved in redox defence, while their fatty-acid composition may influence the susceptibility of circulating and cellular lipids to oxidation. Compared with the direct interaction between topical oils and the tear film, however, the relationship between dietary oil intake and the highly specialised lipid environment of the avascular lens is more indirect. Current cataract-related evidence therefore concerns mainly the maintenance of redox and membrane homeostasis rather than a clearly defined effect of a particular oil on lens transparency.

#### 3.2.3. Retinal and RPE Lipid Homeostasis

The retina has exceptionally high metabolic and lipid requirements. Photoreceptor outer-segment membranes contain abundant polyunsaturated phospholipids and undergo continuous renewal through coordinated lipid uptake, synthesis, transport, recycling, and exchange with the RPE. Membrane lipids contribute to outer-segment organisation and phototransduction, while fatty acids also serve as metabolic substrates and precursors of locally active lipid mediators. The high degree of unsaturation required for photoreceptor function simultaneously increases the susceptibility of the outer retina to photo-oxidation and lipid peroxidation [60].

Natural oils may contribute to retinal lipid nutrition through several routes. Marine and algal oils provide preformed docosahexaenoic acid (DHA) and eicosapentaenoic acid (EPA), whereas plant oils supply α-linolenic acid (ALA), linoleic acid (LA), oleic acid, and other fatty acids in source-specific proportions [61,62]. Carotenoid- and tocopherol-containing oils can also contribute lipid-soluble micronutrients or facilitate their intestinal absorption [25,61]. Lutein and zeaxanthin selectively accumulate in the macula [63,64], whereas vitamin A-derived retinaldehyde is required for the visual cycle [65,66]. Dietary oils may therefore be relevant both as sources of fatty acids and as lipid matrices supporting the absorption and transport of retinally relevant micronutrients.

Retinal lipid metabolism is closely connected with RPE and neurovascular-unit function. The RPE participates in outer-segment phagocytosis, retinoid recycling, lipid transport, cholesterol handling, and maintenance of the outer blood–retinal barrier. Disturbance of these processes may promote lipid accumulation, oxidative injury, complement activation, inflammatory signalling, and impaired photoreceptor support. These events are particularly relevant to AMD, in which extracellular lipid deposition, RPE dysfunction, oxidative stress, and complement activation interact [67,68].

Changes in fatty-acid, cholesterol, and sphingolipid metabolism have also been associated with inflammation and neurovascular-unit dysfunction in DR [67,68]. In inherited retinal degeneration and glaucoma, oxidative stress, glial activation, mitochondrial dysfunction, and altered lipid-mediator signalling may contribute to secondary photoreceptor or retinal ganglion-cell injury [69,70]. The retinal relevance of natural oils therefore extends beyond a single antioxidant pathway and includes membrane composition, lipid transport, energy metabolism, micronutrient delivery, and lipid-mediator generation.

#### 3.2.4. Cross-Tissue Redox and Inflammatory Regulation

Redox and inflammatory regulation provide a common link among the ocular surface, lens, retina, and RPE. These tissues are continually exposed to metabolic oxygen consumption, light, environmental stimuli, and age-related changes in antioxidant capacity. When the generation of reactive oxygen and nitrogen species exceeds local defence systems, membrane lipids undergo peroxidation and generate reactive products that can further modify proteins, nucleic acids, and cellular membranes [41,71].

Oxidative stress and inflammation can reinforce one another. Oxidised lipids, damaged cells, and disrupted barriers may activate epithelial cells, resident immune cells, vascular cells, microglia, and other glial populations. Inflammatory mediators may, in turn, increase mitochondrial stress and reactive-oxygen-species production. The relative importance and cellular location of this cycle differ among DED, uveitis, cataract, AMD, DR, retinal degeneration, and glaucoma [41,71,72].

At the same time, highly unsaturated lipids are susceptible to oxidation during extraction, refining, storage, cooking, and formulation. Depletion of natural antioxidants and accumulation of lipid hydroperoxides, aldehydes, and secondary oxidation products may modify the nutritional and biological properties of an oil [73]. The potential ocular relevance of natural oils is therefore determined not only by their original fatty-acid and micronutrient composition, but also by processing quality, oxidative stability, dose, and route of exposure. Overall, the principal biological links between natural oils and ocular health involve tear-film lipid support, lens membrane and redox homeostasis, retinal and RPE lipid metabolism, micronutrient delivery, and cross-tissue regulation of oxidative and inflammatory responses. This physiological framework provides the context for the source-specific clinical, preclinical, and formulation evidence discussed in subsequent sections.

## 4. Overview of Major Oil-Associated Constituents Relevant to Ocular Health

**Figure 2:**
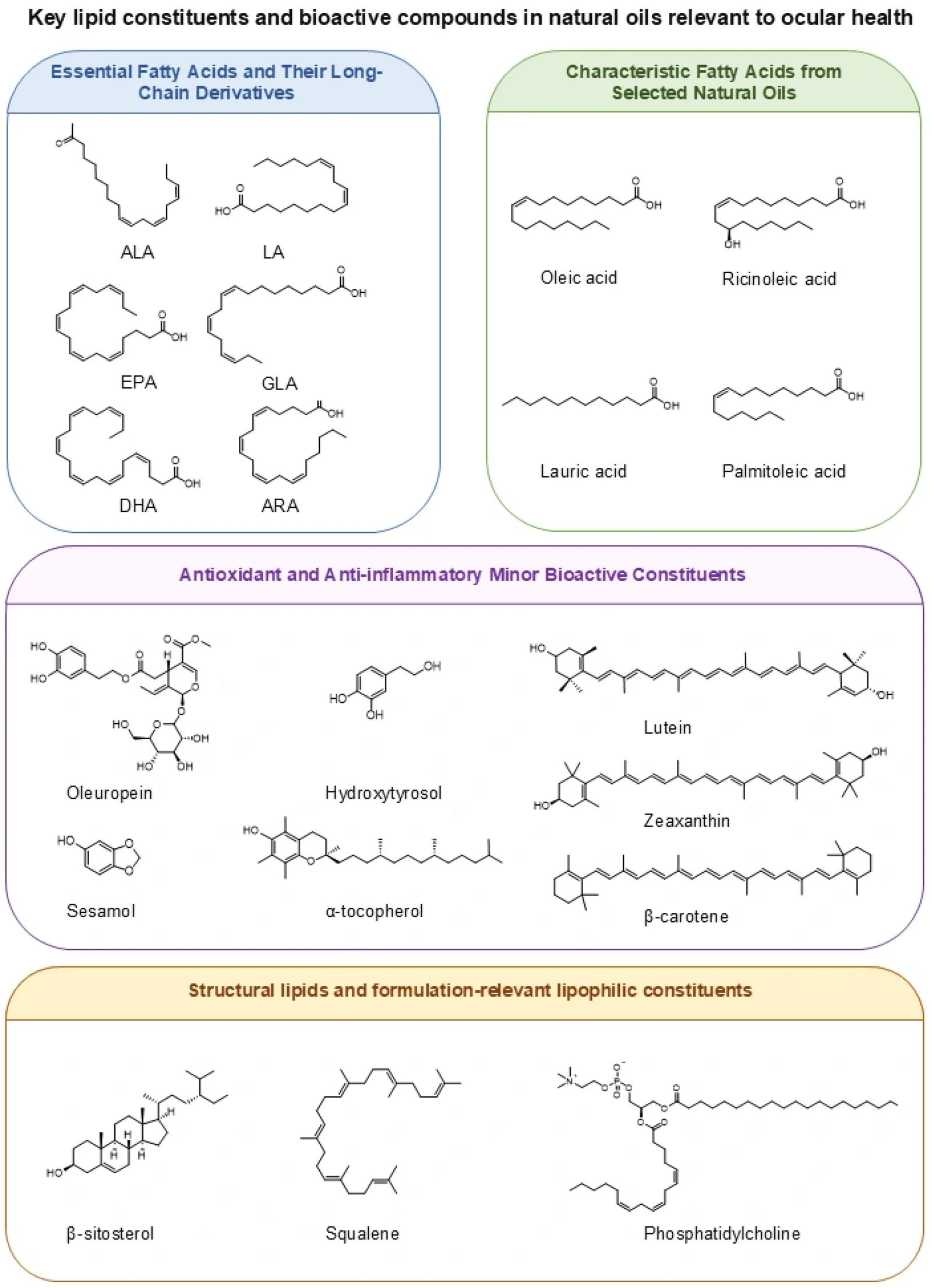
Key lipid constituents and bioactive compounds in natural oils and fats relevant to ocular health. The figure summarises representative fatty acids, minor bioactive constituents, and formulation-relevant lipids found in natural oils and fats. Essential fatty acids and their long-chain derivatives, including ALA, LA, EPA, DHA, GLA, and ARA, are related to membrane structure, lipid-mediator metabolism, and retinal function. Phenolic compounds, tocopherols, and carotenoids may contribute to antioxidant and anti-inflammatory activity, whereas β-sitosterol, squalene, and phosphatidylcholine may influence lipid matrix properties, oxidative stability, and ophthalmic formulation design. ALA, α-linolenic acid; ARA, arachidonic acid; DHA, docosahexaenoic acid; EPA, eicosapentaenoic acid; GLA, γ-linolenic acid; LA, linoleic acid.

Natural oils and fats are multicomponent lipid matrices comprising triacylglycerols (TAGs), fatty acids, phospholipids, phytosterols, phenolic compounds, and, in some oils, carotenoids, fat-soluble vitamins, and other minor constituents [25,61] (Figure 2). These constituents determine the physicochemical properties, oxidative stability, and nutritional value of an oil and may also provide biological plausibility for ocular relevance. Ocular tissues are particularly susceptible to oxidative and lipid-peroxidative injury because of their high metabolic activity, oxygen demand, light exposure, and abundance of oxidisable lipids. Oxidative stress contributes to DED, corneal disorders, cataract, glaucoma, AMD, and DR, while inflammation and immune dysregulation can amplify this injury across ocular–surface, vascular, and neuroretinal compartments [72,74]. Fatty acids and other lipophilic constituents may influence membrane properties, lipid mediator generation, redox balance, inflammatory signalling, and neuronal survival. Omega-3 long-chain polyunsaturated fatty acids (PUFAs), for example, have been investigated in ischaemic, light-induced, inflammatory, and age-related retinal injury [75,76].

The evidence in this section is interpreted at the level of the material actually tested. Findings obtained with an isolated fatty acid, vitamin, carotenoid, phytosterol, phenolic compound, defined constituent combination, or bioactive extract are not treated as evidence that the corresponding whole source oil has the same effect. Likewise, findings from non-ocular cells, systemic tissues, or general metabolic and neurological models are identified as indirect mechanistic evidence rather than proof of ocular efficacy. Whole oil and multi-ingredient evidence is mentioned in this section only where necessary to contextualise constituent-level findings and is examined in greater detail in the source-specific and application sections.

**Figure 3:**
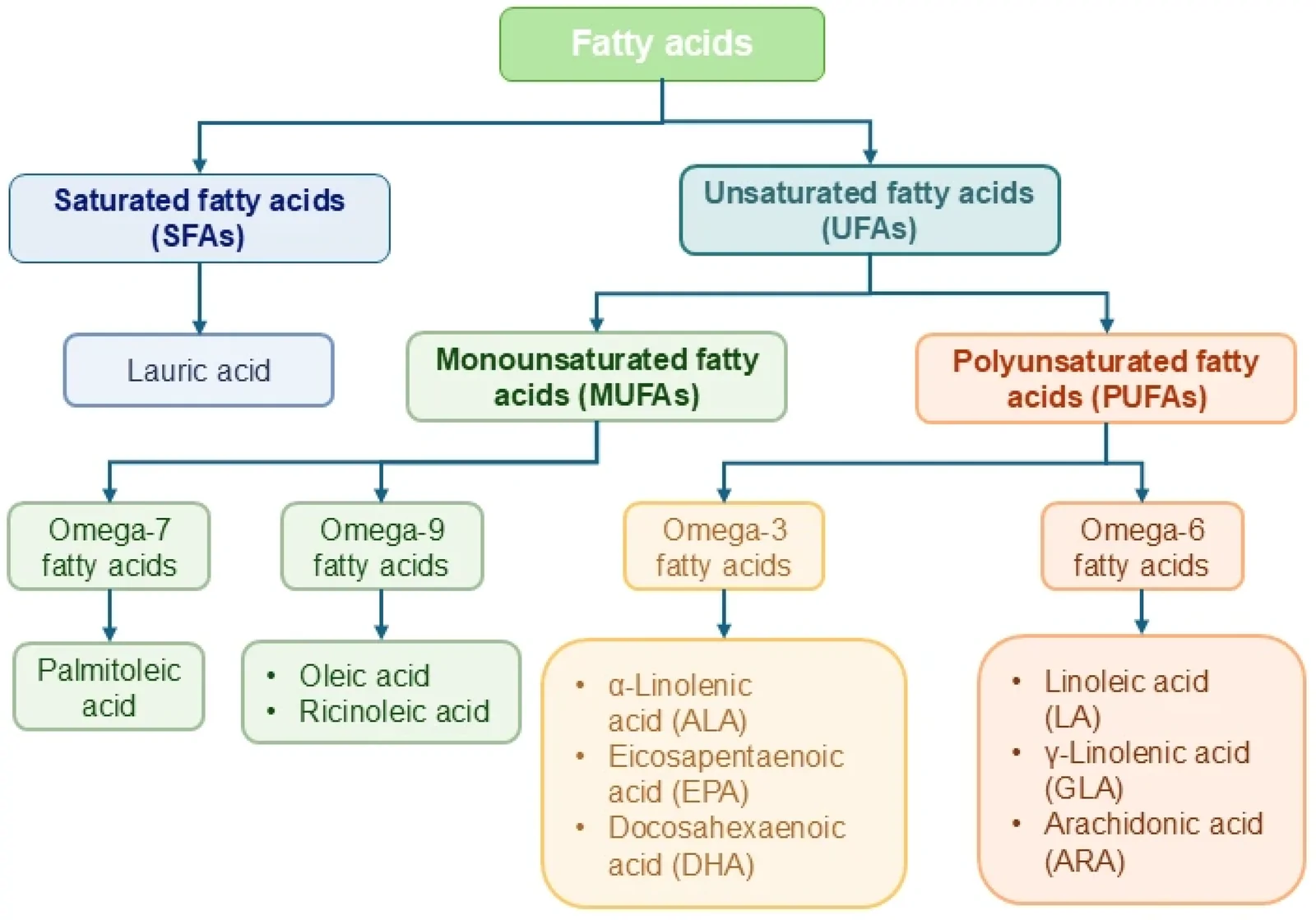
Classification of fatty acids relevant to natural oils and fats. Fatty acids in natural oils and fats can be broadly classified into saturated fatty acids (SFAs) and unsaturated fatty acids. Unsaturated fatty acids are further divided into monounsaturated fatty acids (MUFAs) and polyunsaturated fatty acids (PUFAs). Representative MUFAs include omega-7 fatty acids, such as palmitoleic acid, and omega-9 fatty acids, such as oleic acid and ricinoleic acid. Representative PUFAs include omega-3 fatty acids, such as ALA, EPA, and DHA, and omega-6 fatty acids, such as LA, GLA, and ARA. This classification provides a compositional framework for interpreting the nutritional, biological, and ophthalmic relevance of different natural oils and fats. ALA, α-linolenic acid; ARA, arachidonic acid; DHA, docosahexaenoic acid; EPA, eicosapentaenoic acid; GLA, γ-linolenic acid; LA, linoleic acid.

Fatty acids can be classified by saturation, double-bond position, and carbon-chain length. Saturated fatty acids (SFAs) contain no carbon–carbon double bonds, whereas monounsaturated fatty acids (MUFAs) contain one and PUFAs contain two or more [77]. The principal omega-3 PUFAs are α-linolenic acid (ALA), EPA, and DHA. The principal omega-6 PUFAs include linoleic acid (LA), γ-linolenic acid (GLA), and arachidonic acid (ARA). Oleic acid is a major dietary omega-9 MUFA [78] (Figure 3). In ocular tissues, DHA is enriched in photoreceptor membranes, whereas EPA and ARA act as precursors of lipid mediators. Their ocular relevance therefore depends on the specific fatty acid, chemical form, dose, metabolic context, and balance among competing lipid-mediator pathways.

Fatty acids are also classified as short-chain (SCFAs; C2–C4), medium-chain (MCFAs; C6–C12), and long-chain fatty acids (LCFAs; C14 and above) [79,80]. SCFAs are produced mainly through gut–microbial fermentation of dietary fibre and resistant starch and are not major constituents of most natural oils. Their reported effects on intestinal barrier function and systemic immune homeostasis are therefore indirect to the present topic [81]. MCFAs are absorbed and oxidised relatively rapidly, whereas LCFAs have more prominent roles in membrane architecture, lipid signalling, and neural tissue homeostasis [80,82,83].

Minor oil-associated constituents also require material-specific interpretation. Phenolic acids, flavonoids, oleuropein, and hydroxytyrosol may affect redox and inflammatory pathways, including nuclear factor erythroid 2-related factor 2/haem oxygenase-1 (Nrf2/HO-1) and nuclear factor-kappa B (NF-κB), but studies of purified compounds or phenolic extracts do not establish equivalent effects of the whole oils from which they may be obtained [84]. Similarly, lutein, zeaxanthin, tocopherols, vitamin A, and phytosterols have recognised nutrient- or constituent-level relationships with macular pigment, membrane oxidation, the visual cycle, and lipid metabolism, but these relationships must not be used to infer the clinical efficacy of an untested source oil [85,86,87].

### 4.1. Fatty Acids

#### 4.1.1. Saturated Fatty Acids

SFAs are present in animal fats, dairy products, and selected plant oils and include palmitic acid (C16:0), stearic acid (C18:0), lauric acid (C12:0), and myristic acid (C14:0). As membrane–lipid constituents and energy substrates, they contribute to lipid homeostasis, although their biological effects depend strongly on the dietary and metabolic context [80]. Palmitic and stearic acids have also been identified in retinal and RPE lipids [88].

Human evidence remains observational and inconsistent. An analysis of National Health and Nutrition Examination Survey data did not identify a significant positive association between total SFA intake and AMD, although subgroup trends suggested that broader dietary and metabolic factors may modify the association [89]. Preclinical high-fat-feeding studies have reported altered retinal lipid composition and gene-expression patterns in Müller cells and photoreceptors [90]. In a high-fructose/high-fat mouse model, increased susceptibility to blue-light injury was accompanied by photoreceptor loss, outer-nuclear-layer thinning, blood–retinal barrier disruption, and activation of advanced glycation end product receptor for advanced glycation end product (AGE–RAGE), NF-κB, and inflammasome-related pathways [91].

Overall, SFA-related ocular evidence is limited and context-dependent, and it mainly indicates that an adverse high-fat metabolic environment can modify retinal lipid metabolism and stress susceptibility.

#### 4.1.2. Unsaturated Fatty Acids

##### Monounsaturated Fatty Acids

MUFAs contain one carbon–carbon double bond and include omega-9, omega-7, and less abundant subclasses [92]. Omega-9 MUFAs include oleic, elaidic, gondoic, erucic, and nervonic acids [83], whereas omega-7 MUFAs include palmitoleic and vaccenic acids [93,94]. These fatty acids occur in different proportions in plant oils and animal fats. Evidence from general metabolic and ageing research links MUFA exposure with membrane composition, lipid metabolism, and inflammatory status, but much of this evidence is non-ocular and therefore indirect [95,96,97].

Oleic acid has the most direct ocular evidence among MUFAs, although interpretation remains limited by combined interventions. In an experimental hyperlipidaemia model, a diet containing oleic acid together with long-chain omega-3 fatty acids reduced retinal and systemic inflammatory mediators, downregulated retinal NF-κB-p65, and attenuated acellular capillary formation, pericyte loss, and ganglion cell reduction [98]. Because oleic acid and omega-3 fatty acids were administered together, the observed retinal effects cannot be assigned to oleic acid alone or to an oleic acid-rich whole oil. In a 2024 case-control study of people with type 2 diabetes, higher circulating MUFA and oleic acid levels were inversely associated with DR, but the observational design does not establish causality [99]. Cell-based studies further indicate that fatty-acid species can alter inflammatory mediator release from retinal Müller cells, with effects depending on concentration and metabolic conditions [100].

For AMD, epidemiological evidence for total MUFA intake is inconsistent and generally neutral or weakly inverse [89]. Direct ocular intervention evidence for palmitoleic, vaccenic, gondoic, erucic, and nervonic acids is sparse. Changes in palmitoleic and vaccenic acids have been reported in retinal lipid profiles after high-fat feeding [101], whereas associations involving vaccenic, gondoic, and erucic acids arise mainly from brain-ageing or general neuro-metabolic research [102,103]. Nervonic acid has protected non-ocular neuronal and Schwann cell models from oxidative injury and promoted peripheral nerve-regeneration markers in rats, but these findings are indirect and cannot establish retinal efficacy [104]. Associations between MUFA status, metabolism, and low-grade inflammation in older adults are likewise relevant only as systemic biological context [105,106,107].

Thus, oleic acid has limited ocular observational and preclinical support, while evidence for other MUFAs is predominantly indirect.

##### Polyunsaturated Fatty Acids

PUFAs include the omega-3 series, principally ALA, EPA, and DHA, and the omega-6 series, principally LA, GLA, and ARA. ALA and LA are essential fatty acids that must be obtained from the diet [78]. PUFAs occur in varying proportions in seed oils, marine oils, and concentrated nutritional supplements [62].

Most nutritionally relevant PUFAs are LCFAs. Their intestinal absorption requires lipolysis, incorporation into mixed micelles, enterocyte uptake, re-esterification, and transport mainly in chylomicrons [108]. These processes are relevant because TAG, phospholipid, free-fatty-acid, and ethyl-ester forms can differ in digestion and bioavailability, but detailed gastrointestinal physiology is outside the ophthalmic focus of this review.

##### Omega-3 Fatty Acids

Omega-3 PUFAs include plant-derived ALA and the long-chain fatty acids EPA and DHA [78]. Conversion of ALA to EPA and especially DHA is limited, whereas fish, krill, and microalgal products can provide preformed EPA and/or DHA. Their bioavailability varies with chemical form, including TAG, phospholipid, free-fatty-acid, and ethyl-ester forms [109].

EPA and DHA compete with ARA for cyclooxygenase, lipoxygenase, and cytochrome P450 pathways and alter the production of prostaglandins, leukotrienes, and related lipid mediators. EPA can generate E-series resolvins, while DHA can generate D-series resolvins, protectins, neuroprotectin D1, and maresins [110]. General metabolic and neurological studies also show effects on triglyceride metabolism, systemic inflammatory status, and neuronal–membrane composition, but these non-ocular findings provide only indirect context for ocular hypotheses [111,112].

On the ocular surface, topical or oral omega-3 interventions have been investigated in relation to tear-film stability, epithelial injury, and inflammatory signalling. DED is associated with tear hyperosmolarity, epithelial damage, and increased inflammatory mediators, including IL-1β, IL-6, IL-8, IL-17A, interferon-γ, and tumour necrosis factor-α [113]. A systematic review of topical omega-3 formulations reported improvements in selected human tear-film and staining outcomes, while animal studies showed reductions in cytokine expression, monocyte infiltration, epithelial injury, and goblet-cell loss [114]. Despite this biological rationale, clinical findings are heterogeneous. The large DREAM trial did not demonstrate superiority of oral omega-3 supplementation over placebo for DED symptoms or major objective signs [115] (Table 1). Its inflammatory-marker substudy identified associations between changes in systemic inflammatory markers and changes in selected ocular-surface outcomes [116].

**Table 1:** Human clinical and observational evidence.

Condition	Tested Material/Category	Design/Population (*n*)	Comparator	Dose/Route/Duration	Ocular Endpoint(s)	Main Finding	Evidence Interpretation	Ref.
DED	EPA/DHA concentrate; isolated fatty acids	DREAM; large multicentre RCT; moderate-to-severe DED; *n* = 535	Olive-oil placebo	Oral EPA 2 g/day + DHA 1 g/day; 12 months	OSDI; staining; TBUT; Schirmer test	No superiority over placebo for DED signs or symptoms	Large RCT; neutral result	[115]
DED	OTX-101 0.09%; cyclosporine nanomicelles	Phase III multicentre double-masked RCT; *n* = 744	Vehicle	Topical twice daily; 84 days	≥10-mm Schirmer gain; staining; symptoms; safety	More Schirmer responders; improved staining; no symptom difference	Product-level evidence	[117]
DED	OM3 artificial tear; flaxseed/castor oils, CMC and glycerin; multi-ingredient	Multicentre double-masked RCT; *n* = 242	Refresh Optive Advanced® (Allergan, Inc., Irvine, CA, USA) ^®^	Topical OM3; 90 days	OSDI; staining; TBUT; safety	Both groups improved; OM3 superior for selected staining outcomes	Product level; mixed results	[118]
DED	EPA/DHA capsules; concentrated fatty acids	Double-masked placebo-controlled RCT; 518 eyes	Placebo capsules	Oral EPA 650 mg/day + DHA 350 mg/day; 3 months	TBUT; symptoms; Schirmer test	Greater improvements in TBUT, symptoms and Schirmer test	3-month RCT; outcomes reported by study eye	[119]
DED; cold-season osmolarity	Sea buckthorn oil; whole oil	Double-blind placebo-controlled RCT; *n* = 100; 86 completers	Placebo oil	Oral SBO 2 g/day; 3 months	Tear osmolarity; TBUT; redness; burning	Reduced seasonal osmolarity increase and selected symptoms	Outcome-specific benefit	[120]
Contact lens-associated dry eye	Evening primrose oil; whole oil	Double-masked placebo-controlled RCT; women, *n* = 76	Olive-oil placebo	Oral GLA 300 mg/day; 6 months	Symptoms; comfort; tear meniscus; TBUT; MGD signs; staining	Improved symptoms, comfort and tear meniscus; most signs unchanged	Symptomatic benefit; limited objective change	[121]
DED	Krill oil or fish oil; whole marine oils	Double-masked placebo-controlled RCT; *n* = 60; 54 completers	Olive-oil placebo	Krill: EPA 945 + DHA 510 mg/day; fish: EPA 1 g + DHA 500 mg/day; 90 days	Osmolarity; OSDI; TBUT; redness; tear IL-17A	Reduced tear osmolarity with both oils vs. placebo	Product-specific RCT	[122]
DED with MGD	DHA-dominant EPA/DHA supplement; concentrated fatty acids	Double-masked placebo-controlled RCT; *n* = 50	Olive-oil placebo	Oral EPA 600 mg + DHA 1.64 g/day; 8 weeks	MGD score; TBUT; staining; meniscometry; OSDI	Greater TBUT and MGD-score improvement vs. placebo	Small, short RCT	[123]
Sjögren-associated dry eye	Flaxseed oil; whole oil	RCT; women with Sjögren-associated disease; *n* = 38	Placebo	Oral flaxseed oil 1 or 2 g/day; 180 days	Symptoms; inflammation; Schirmer I; TBUT	Reduced inflammation and symptoms; improved Schirmer I and TBUT	Small, disease-specific RCT	[124]
Anterior blepharitis; MGD	100% cold-pressed castor oil; whole oil	Investigator-masked paired-eye RCT; *n* = 26	Untreated fellow eye	Periocular twice daily; 4 weeks	Lid/lash signs; lid-wiper epitheliopathy; symptoms; safety	Improved several lid/lash signs and OSDI; no adverse events	Small, short paired-eye RCT	[125]
DED; inflammation	LA + GLA; isolated fatty acids	RCT; aqueous-deficient DED; *n* = 26	Placebo tablet + tear substitute	Oral LA 28.5 mg + GLA 15 mg twice daily; tear substitute; 45 days	Symptoms; staining; inflammation; FBUT; Schirmer I	Improved symptoms, staining and inflammation; unchanged FBUT and Schirmer I	Small, 45-day RCT	[126]
DED	Fish-oil EPA/DHA capsules; concentrated fatty acids	Placebo-controlled RCT; DED; *n* = 27	Placebo capsules	Oral EPA 1.245 g + DHA 540 mg/day; 12 weeks	Eye pain; TBUT; Schirmer I; fluorescein and rose bengal staining	Improved DED parameters vs. placebo	Small, short RCT	[127]
DED	EPA/DHA capsules; concentrated fatty acids	Double-blind RCT; DED; *n* = 64	Medium-chain triglyceride-oil capsules	Oral EPA 360 mg + DHA 240 mg/day; 30 days	TBUT; Schirmer test; OSDI; tear evaporation	Reduced evaporation and symptoms; improved tear secretion	Short-term RCT	[128]
MGD; evaporative DED	2% castor-oil emulsion; multi-ingredient	Double-masked placebo-controlled crossover; *n* = 20; 40 eyes	Vehicle	Topical six times/day; two 2-week periods	Symptoms; interference grade; evaporation; staining; TBUT; orifice obstruction	Improved symptoms, evaporation, staining, TBUT and obstruction	Small crossover RCT	[129]
DED; lipid-layer deficiency	Castor-oil emulsion; multi-ingredient	Open-label pilot; healthy *n* = 5; DED *n* = 10	Baseline	Single topical instillation; 4 h	Tear retention; lipid layer; symptoms	Retention up to 4 h; transient lipid-layer and symptom improvement	Uncontrolled pilot	[130]
Multiple DED subtypes	Phospholipid nanoemulsion lubricant; multi-ingredient	Prospective multicentre single-arm study; *n* = 70	Baseline	Topical twice daily; 28 days	OSDI; TBUT; staining; VA; tolerability	Improved TBUT and staining; no significant adverse events	Uncontrolled product-level evidence	[131]
DED with MGD	Lipid nanoemulsion artificial tears; multi-ingredient	Retrospective cohort; 100 eyes	Systane Balance artificial tears	Topical; 3 months	NITBUT; Schirmer I; staining; gland loss; symptoms	Longer NITBUT; greater staining and symptom improvement	Retrospective association	[132]
DED	Optive Plus® (Allergan, Inc., Irvine, CA, USA); castor oil, CMC and glycerin; multi-ingredient	Prospective multicentre real-world study; *n* = 1209	None	Topical; 4 weeks	Signs; symptoms; tolerability; safety	Improved signs and symptoms; generally well tolerated	Uncontrolled product-level evidence	[133]
Evaporative DED	Emustil® (SIFI S.p.A., Catania, Italy); soybean oil and phospholipids; multi-ingredient	Randomised comparative trial; lipid-deficient evaporative DED; *n* = 75	HA and HPMC tears	Topical four times daily; 90 days	Symptoms; evaporation; stability; osmolarity; staining	Greater improvement in stability, osmolarity and staining	Product-level evidence	[134]
Low-humidity discomfort	Emustil® (SIFI S.p.A., Catania, Italy); soybean oil and phospholipids; multi-ingredient	Controlled low-humidity model; healthy men, *n* = 12	Exposure/timing comparison	Topical before or after exposure; acute	Lipid-layer thickness; NITBUT; tear evaporation	Preventive use improved lipid layer and NITBUT; other modes improved selected outcomes	Healthy volunteers; acute product-level evidence	[135]
Low-humidity discomfort	Emustil® (SIFI S.p.A., Catania, Italy); multi-ingredient emulsion	Controlled low-humidity model; healthy participants; *n* = 12	Exposure/timing comparison	Topical before or after exposure; acute	Discomfort; tear osmolarity; PRT	Improved selected tear-film parameters	Environmental-stress product evidence	[136]
Dry eye symptoms/ocular surface discomfort	SBO/hyaluronate eyelid spray; multi-ingredient	Multipart RCT; symptomatic adults aged 25–70 years	Reference spray or untreated fellow eye	Closed-eyelid spray; 1 day, 9 days or 1.5 months	OSDI; symptoms; TBUT; osmolarity; PRT; tolerability	Reduced OSDI, dryness and watering; unchanged TBUT and osmolarity	Symptomatic product-level benefit	[137]
Self-reported ocular dryness	Omegia™ sea buckthorn oil softgel (Puredia Corporation Limited, Hong Kong, China); whole oil	Placebo-controlled RCT; women aged ≥ 45 years	Placebo softgel	Oral 500 mg/day; 12 weeks	Ocular symptoms; catalase; TNF-α	Improved self-rated symptoms; increased catalase; reduced TNF-α	Questionnaire-based preliminary evidence	[138]
Meibomian gland dysfunction	Omega-3/antioxidant supplement; multi-ingredient	Double-masked placebo-controlled RCT; MGD; *n* = 61	Placebo; identical lid hygiene and artificial tears	Oral omega-3 1.5 g/day; 3 months	OSDI; TBUT; lid inflammation; gland expression; Schirmer I	Improved multiple outcomes from baseline	Small, short product-level RCT	[139]
AMD progression	DHA/EPA added to AREDS; combined formulation	Multicentre double-masked AREDS2 RCT	AREDS formulation	Oral DHA 350 + EPA 650 mg/day; long-term follow-up	Late AMD progression; vision; safety	No additional reduction in AMD progression	Large RCT; neutral result	[140]
AMD	Habitual olive-oil intake; observational whole-oil exposure	Alienor population cohort; *n* = 654; 1269 eyes	No use	Regular dietary use; no defined dose	Early and late AMD	Lower late-AMD risk; no association with early AMD	Observational association	[141]
Macular-pigment nutrition	Whole avocado; whole-food lipid matrix	Six-month RCT; older adults, *n* = 40	One potato or one cup of chickpeas per day	One avocado/day; 6 months	Serum lutein; MPD	Serum lutein +25%; increased MPD	Whole-food context; not direct oil evidence	[142]
Infant vision	Graded algal-DHA formulas; multi-ingredient	Double-masked RCT; term infants, *n* = 343	0% DHA formula	DHA 0%, 0.32%, 0.64% or 0.96%; infancy	12-month visual acuity; RBC fatty acids; tolerability; safety	0.32% DHA improved visual acuity; no added benefit at higher doses	Formulation-level evidence	[143]
Infant vision	Maternal high-DHA algal-oil supplement	Randomised supplementation trial; lactating women and infants	DHA-free vegetable oil	Maternal oral DHA ~200 mg/day; 4 months postpartum	Milk/plasma DHA; infant vision; development	Improved DHA status; no consistent infant visual-function benefit	Maternal-supplement evidence	[144]
Infant growth and DHA status	Infant formula containing DHASCO® or DHASCO®-B algal DHA (DSM Nutritional Products, Columbia, MD, USA)	Double-blind RCT; term infants; *n* = 140 vs. 127	Commercial formula containing DHASCO® algal DHA	Formula DHA 17 mg/100 kcal; 120 days	Growth; tolerability; adverse events; RBC DHA	Comparable growth, tolerability and RBC DHA status	Nutritional equivalence; no ocular efficacy endpoint	[145]

Notes: AMD, age-related macular degeneration; AREDS, Age-Related Eye Disease Study; AREDS2, Age-Related Eye Disease Study 2; CMC, carboxymethylcellulose; DED, dry eye disease; DHA, docosahexaenoic acid; DHASCO, docosahexaenoic acid-rich single-cell oil; DREAM, Dry Eye Assessment and Management; EPA, eicosapentaenoic acid; FBUT, fluorescein breakup time; GLA, γ-linolenic acid; HA, hyaluronic acid; HPMC, hydroxypropyl methylcellulose; IL-17A, interleukin-17A; LA, linoleic acid; MGD, meibomian gland dysfunction; MPD, macular pigment density; NITBUT, non-invasive tear breakup time; OSDI, Ocular Surface Disease Index; PRT, phenol red thread test; RBC, red blood cell; RCT, randomised controlled trial; SBO, sea buckthorn oil; TBUT, tear breakup time; TNF-α, tumour necrosis factor-α; VA, visual acuity.

DHA-derived lipid mediators have also been studied in corneal-nerve repair. Preclinical models indicate that neuroprotectin D1 can promote corneal-nerve recovery after surgical injury, and dietary omega-3 interventions have been associated with reduced diabetes-related corneal nerve loss and enhanced regeneration [52]. This evidence is biologically relevant but remains predominantly preclinical.

In the retina, DHA is an abundant structural component of photoreceptor outer-segment membranes and contributes to membrane fluidity, rhodopsin signalling, and disc renewal [88]. Experimental studies suggest that EPA/DHA can modulate reactive oxygen species, lipid peroxidation, glutathione-related defence, NF-κB-associated inflammation, and VEGF/hypoxia-inducible factor-1α-related angiogenic signalling [146]. In DR, oxidative and vascular injury involves mitochondrial reactive oxygen species, AGE–RAGE and protein kinase C signalling, impaired Nrf2/sirtuin 1 activity, lipid peroxidation, and ferroptosis-related pathways [147]. A combined analysis of the Multi-Ethnic Study of Atherosclerosis and Genetics of Latino Diabetic Retinopathy cohorts found that circulating DHA, but not EPA, was inversely associated with DR presence and severity [148].

For AMD, omega-3-related hypotheses involve photoreceptor lipid composition, RPE oxidative injury, complement activation, and choroidal angiogenesis [88]. Prospective and genetic analyses have associated higher circulating omega-3 PUFAs, particularly DHA, with a lower risk of AMD [149]. However, AREDS2 did not demonstrate an additional benefit when DHA/EPA was added to the background AREDS formulation [140] (Table 1).

For retinitis pigmentosa and other inherited retinal degenerations, the rationale for DHA supplementation is based mainly on photoreceptor structure and experimental neuroprotection. A 2025 American Academy of Ophthalmology assessment concluded that available supplementation studies were limited by small samples, heterogeneous endpoints, and post-hoc analyses and did not establish that DHA or other dietary supplements delay disease progression [150].

Overall, omega-3 PUFAs have strong structural and mechanistic relevance to ocular tissues, but their clinical effects are disease-, formulation-, and route-specific. Supportive findings from smaller trials, observational studies, and preclinical models should therefore be interpreted in the context of neutral findings from higher-quality clinical evidence.

##### Omega-6 Fatty Acids

Omega-6 PUFAs include LA, GLA, dihomo-γ-linolenic acid, and ARA. LA is essential, while GLA and dihomo-γ-linolenic acid are intermediates in the conversion pathway towards ARA [151,152]. Dietary sources include sunflower, corn, soybean, sesame, and cottonseed oils, as well as animal-derived foods [153]. ARA is metabolised through cyclooxygenase and lipoxygenase pathways to prostaglandins, leukotrienes, and thromboxanes. Although these mediators participate in inflammation and vascular regulation, general epidemiological evidence does not support classifying all omega-6 PUFAs as uniformly pro-inflammatory or harmful [154,155].

ARA is present in corneal, vitreous, and retinal phospholipids; comparative tissue analyses have reported relatively high retinal ARA proportions in sheep and camels, providing a structural substrate for lipid-mediator production [156].

Human ocular-surface evidence is derived mainly from GLA-containing or mixed essential-fatty-acid supplements. Some randomised trials reported improvement in subjective DED symptoms, whereas objective signs were inconsistent [157] (Table 1). A recent review similarly concluded that the evidence for symptom improvement remains uncertain, although selected clinical signs may benefit [158].

Evidence relating omega-6 PUFAs to cataract is inconsistent. Reviews have linked overall PUFA exposure with cataract risk without adequately isolating omega-6 fatty acids [159], while an earlier observational study associated high LA intake with nuclear lens opacity [160]. UVB cataract models have implicated prostaglandin E2 and prostaglandin F2α in lens injury [161], and LA can alter connexin-46 hemichannel function in experimental lens systems [162].

In retinal disease, ARA-derived prostaglandin E2, thromboxane A2, and leukotriene B4 can participate in inflammatory, vascular, and oxidative responses [163,164]. However, epidemiological evidence does not support a simple interpretation that higher absolute omega-6 intake independently increases AMD risk. A recent meta-analysis found no consistent association between higher intake of omega-6 PUFAs, LA, or ARA and the risk of overall, early, or intermediate AMD [165], whereas population-based evidence from Korean adults suggested that a higher dietary omega-6/omega-3 ratio may be associated with increased AMD prevalence in women [166]. Observational and Mendelian-randomisation studies have also reported inverse associations between selected circulating omega-6 PUFA measures and DR risk [167,168,169].

Developmental and neovascular-retinal evidence further illustrates the importance of formulation and fatty-acid balance. Combined ARA and DHA supplementation was associated with a lower risk of severe retinopathy of prematurity in extremely preterm infants, while preclinical maternal DHA-plus-ARA interventions supported physiological retinal vascular growth and mitochondrial metabolism in offspring exposed to hyperglycaemic stress [170,171]. In oxygen-induced retinopathy models, lowering the omega-6/omega-3 ratio reduced pathological neovascularisation and microglial activation [172].

Overall, omega-6 PUFAs serve both structural and signalling functions in ocular tissues. Their effects vary by fatty-acid species, metabolite profile, disease context, and balance with omega-3 PUFAs; current evidence does not support a simple protective-versus-harmful classification.

### 4.2. Phenolic Compounds

Phenolic compounds are minor constituents of many plant oils and include phenolic acids, flavonoids, lignans, secoiridoids, and simple phenols. Although present at relatively low concentrations, they can influence oil oxidative stability and biological activity through hydrogen or electron donation, radical scavenging, metal chelation, and interruption of lipid-peroxidation reactions [84,173]. Their abundance depends strongly on plant source, maturity, extraction, refining, heating, and storage [61,174].

Compared with fatty acids, which mainly participate in membrane-lipid composition and lipid-mediator generation, phenolic compounds are more commonly considered to influence ocular disease processes by regulating oxidative stress, inflammatory signalling, pathological angiogenesis, apoptosis, and neuroprotection-related pathways [175].

As the first barrier of the ocular surface, the cornea is vulnerable to ultraviolet radiation, dryness, infection, and mechanical irritation. Relevant reviews have suggested that phenolic compounds can influence corneal inflammation, corneal wound repair, corneal neovascularisation, and dry-eye-related changes in in vivo and in vitro models through antioxidant, anti-inflammatory, antimicrobial, and epithelial-repair-promoting effects [176].

Their anti-angiogenic effects may be related to downregulation of pro-angiogenic factors such as VEGF and HIF-1α. They may also inhibit the expression and activity of matrix metalloproteinases (MMPs), thereby reducing extracellular matrix degradation, cell migration, and epithelial–mesenchymal transition, and consequently influencing pathological processes such as corneal scarring, fibrosis, and abnormal neovascularisation [177].

At the molecular level, phenolic compounds can enhance cellular resistance to oxidative injury by activating antioxidant pathways such as Nrf2/HO-1, while also inhibiting NF-κB, mitogen-activated protein kinase (MAPK), signal transducer and activator of transcription 3 (STAT3), VEGF, and inflammasome-related signalling. These actions may reduce the expression of inflammatory mediators such as TNF-α, IL-1β, IL-6, cyclooxygenase-2 (COX-2), and inducible nitric oxide synthase (iNOS), thereby attenuating ocular tissue injury jointly driven by chronic inflammation and oxidative stress [75,178]. Most of this evidence remains cellular or preclinical.

For AMD, flavonoid-related hypotheses involve oxidative injury, inflammation, mitochondrial dysfunction, complement activity, and choroidal neovascularisation [179,180]. For DR, phenolic compounds have been investigated against hyperglycaemia-associated reactive oxygen species, AGE accumulation, NF-κB activation, vascular endothelial growth factor expression, and blood–retinal barrier disruption [147,181]. Most of these findings arise from cellular and animal studies. Clinical syntheses of flavonoid supplementation suggest possible benefits for selected ocular outcomes, particularly with flavan-3-ol- or anthocyanin-containing interventions, but substantial heterogeneity remains in the compounds, formulations, doses, diseases, and endpoints studied [182,183].

### 4.3. Phytosterols

Phytosterols are plant sterols structurally related to cholesterol. Common forms in foods and oils include β-sitosterol, campesterol, stigmasterol, brassicasterol, and avenasterol [184,185].

Natural oils and fats are important dietary sources of phytosterols. Common phytosterols in plant-derived foods and oils include β-sitosterol, campesterol, stigmasterol, brassicasterol, and avenasterol, among which β-sitosterol is usually one of the most abundant and widely studied forms [186]. They occur as free sterols, esters, glycosides, and acylated glycosides, and their abundance is affected by plant source, extraction, refining, and storage [187]. Neutralisation, deodorization, and high-temperature processing can reduce phytosterol content or generate phytosterol oxidation products [188,189].

The best-established physiological effect of phytosterols is reduced intestinal cholesterol absorption and lower low-density lipoprotein cholesterol; proposed antioxidant, anti-inflammatory, and immunometabolic effects are broader but less directly established [190,191]. Retinal cholesterol and sterol homeostasis is relevant to AMD, DR, and retinal vascular function [192], but systemic effects on cholesterol absorption, lipoproteins, or inflammation represent indirect evidence for ocular health [20,193]. General mechanistic studies showing inhibition of reactive oxygen species, inducible nitric oxide synthase, and the NF-κB–cyclooxygenase-2–prostaglandin E2 axis likewise provide plausibility rather than direct ocular efficacy [20].

Preclinical studies of isolated stigmasterol provide more direct ocular evidence. A 2025 integrative study combining computational analyses and animal experiments associated stigmasterol with improved glucolipid metabolism, systemic inflammation, and ocular vascular changes, potentially through peroxisome proliferator-activated receptor-γ regulation [194]. A separate study reported that stigmasterol reduced reactive oxygen species, apoptosis, and autophagy/mitophagy-related injury in high-glucose-exposed ARPE-19 cells and mouse DR models, possibly through vesicle-associated membrane protein 7-related signalling [195].

Overall, phytosterol-related ocular evidence remains predominantly preclinical or indirect, with insufficient human intervention evidence to support an established ophthalmic effect [196,197].

### 4.4. Fat-Soluble Vitamins

Fat-soluble vitamins are also important non-fatty-acid-accompanying constituents in natural oils and fats, mainly including vitamins A, D, E, and K. Among them, vitamin E is the most representative in oils and fats and usually comprises homologues such as tocopherols and tocotrienols. α-Tocopherol is the principal form that meets human vitamin E requirements [25,198]. Tocopherols occur widely in plant oils, whereas tocotrienols are particularly characteristic of palm oil [87,199]. As a lipophilic chain-breaking antioxidant, α-tocopherol can interrupt PUFA peroxidation, providing a mechanistic basis for studies of retinal, lens, and vascular lipid injury [200,201].

#### 4.4.1. Vitamin E

In patients with type 1 diabetes, high-dose vitamin E supplementation was reported to normalise abnormal retinal blood flow independently of glycaemic control [202]. In diabetic rats, d-α-tocopherol attenuated retinal blood-flow abnormalities, diacylglycerol accumulation, and protein kinase C activation [203].

AREDS evaluated vitamin E only as part of a combined antioxidant and mineral formulation; it therefore does not establish an independent therapeutic effect of vitamin E [204]. For cataract, observational meta-analyses associate higher dietary or circulating vitamin E with lower risk [205,206], whereas randomised trials, including the Physicians’ Health Study II, did not establish a significant preventive effect of vitamin E alone.

#### 4.4.2. Vitamin A

Vitamin A has a direct physiological role in vision. The chromophore 11-cis-retinal is converted to all-trans-retinal during phototransduction and regenerated through the RPE-dependent visual cycle [65,66]. Vitamin A deficiency can cause impaired dark adaptation, night blindness, conjunctival and corneal xerosis, keratomalacia, and irreversible visual impairment [207]. These established deficiency-related effects do not imply that additional vitamin A benefits vitamin A-replete individuals or all retinal diseases. Responses in inherited retinal degeneration and visual-cycle disorders depend on genotype, retinaldehyde metabolism, dose and co-administered nutrients, and excessive exposure can be harmful [208,209].

#### 4.4.3. Vitamin D

Vitamin D has been studied mainly in relation to ocular–surface immune regulation and DED. Meta-analyses suggest that supplementation may improve symptoms and selected clinical signs in some vitamin D-deficient or DED populations, but optimal dosing, route, and target groups remain uncertain [210]. Trials of combined products containing vitamin D3, lutein, zeaxanthin, and curcumin reported improvements in selected dry-eye outcomes, but these results apply to the complete formulation and cannot be attributed to vitamin D alone [211,212]. Vitamin D should therefore be described as a potentially relevant nutritional or immune-regulatory factor rather than an established stand-alone DED therapy [213,214].

#### 4.4.4. Vitamin K

Vitamin K evidence is predominantly mechanistic and preclinical. Cell and animal studies suggest that vitamin K can limit RPE ferroptosis through vitamin K epoxide reductase/ferroptosis suppressor protein 1 signalling and modulate the eukaryotic initiation factor 2α-activating transcription factor 4–vascular endothelial growth factor A axis, linking lipid-peroxidative cell death with angiogenic signalling [215,216]. These findings do not yet support clinical supplementation recommendations or efficacy claims for vitamin-K-containing oils.

### 4.5. Carotenoids

Carotenoids are a class of lipophilic natural pigments mainly synthesised by plants, algae, and some microorganisms. Structurally, they usually contain an isoprenoid backbone with multiple conjugated double bonds, which enables visible-light absorption and participation in redox reactions [217,218]. According to structural differences, carotenoids can be divided into oxygen-free carotenes and oxygen-containing xanthophylls. The former include α-carotene, β-carotene, and lycopene, whereas the latter include lutein, zeaxanthin, cryptoxanthin, and astaxanthin [217,218]. Some carotenoids, such as β-carotene, α-carotene, and β-cryptoxanthin, are provitamin A carotenoids and can be converted in vivo into retinol and its derivatives. By contrast, lutein, zeaxanthin, lycopene, and astaxanthin cannot be converted into vitamin A.

Natural oils can act as dietary matrices or carriers for carotenoids. Red palm oil contains α- and β-carotene and other pigments, while tomato-seed, sea-buckthorn, rapeseed, and flaxseed oils can contain smaller and source-dependent carotenoid fractions [219].

Lutein, zeaxanthin, and meso-zeaxanthin selectively accumulate in the macula, where they contribute to macular pigment, short-wavelength light filtration, and antioxidant defence [63,64]. Systematic reviews and network meta-analyses indicate that antioxidant nutritional formulations can increase macular pigment optical density and influence selected visual outcomes, but effects differ among formulations [76].

AREDS2 provides formulation-level evidence. Its primary analyses did not show a significant additional reduction in progression to late AMD after adding lutein/zeaxanthin or DHA/EPA to the background AREDS formulation [140] (Table 1). Longer-term comparisons supported replacing β-carotene with lutein/zeaxanthin within that formulation [220].

For DR, reviews and animal studies indicate that lutein and zeaxanthin can modulate oxidative stress, inflammatory signalling, and retinal neurovascular injury [214,221]. These findings are mainly preclinical. For cataract, observational meta-analyses associate higher lutein/zeaxanthin intake with lower risk, whereas the AREDS2 cataract trial did not reduce cataract surgery or progression in the overall population; a possible signal was restricted to participants with lower baseline dietary intake [222,223].

Astaxanthin is an oxygenated carotenoid obtained principally from algae and marine organisms. Experimental reviews describe antioxidant, anti-inflammatory, and microcirculatory mechanisms across several ocular models [224]. However, the cited human studies used multi-ingredient test foods. One combined anthocyanins, astaxanthin, and lutein and reported selected accommodative and pupillary-response improvements after visual-display terminal loading [225]. Another combined astaxanthin with tocotrienols and reported improvements in selected functional visual measures [226]. Both outcomes must be attributed to the tested product rather than to astaxanthin alone or to an astaxanthin-containing oil.

Overall, although lipophilic vitamins and carotenoids are mostly minor accompanying constituents in natural oils and fats, their roles in antioxidant defence, photoprotection, the visual cycle, immune regulation, and lipid-peroxidation control make them an important active basis linking the nutritional properties of natural oils and fats with their potential relevance to ocular health.

### 4.6. Other Minor Lipophilic Constituents

Phospholipids, squalene, coenzyme Q, chlorophyll-related compounds, and triterpenoids may occur in selected oils or oil-associated fractions. Their abundance depends on source, extraction, refining, and storage [30,34]. Because these materials differ markedly in biological and pharmaceutical function, they are considered separately below [36].

#### 4.6.1. Phospholipids

Phospholipids are membrane constituents and amphiphilic formulation materials. Polar lipids at the interface between aqueous and non-polar tear components may support lipid spreading and tear-film stability, providing relevance to meibomian-gland dysfunction and evaporative DED [227,228]. However, evidence from egg-derived phospholipids in multi-ingredient eye drops must be interpreted at the product level.

Phospholipid binding can also modify fatty-acid delivery. In animals, lysophosphatidylcholine-DHA increased retinal DHA more effectively than TAG-DHA or free DHA, highlighting the relevance of molecular delivery form to retinal uptake [229].

#### 4.6.2. Squalene

Squalene is a triterpene hydrocarbon present in olive and amaranth oils and in human sebum [230,231]. Nuclear magnetic resonance studies have detected squalene in tears and examined its association with meibomian-lipid film properties, suggesting possible relevance to the ocular–surface lipid environment [232].

#### 4.6.3. Coenzyme Q

Coenzyme Q10 participates in mitochondrial electron transport and endogenous antioxidant defence. Reviews and experimental studies describe potential antioxidant and neuroprotective roles in glaucoma, AMD, and retinitis pigmentosa, although the evidence remains predominantly mechanistic, preclinical, or early clinical [233,234].

#### 4.6.4. Chlorophyll

Chlorophyll and chlorophyll-derived pigments may occur as minor components in oils obtained from green plant materials [30,61]. More direct ophthalmic evidence concerns engineered chlorophyll-derived photosensitisers rather than dietary chlorophyll [235,236]. Mono-L-aspartyl chlorin e6 and related chlorin derivatives have been evaluated for photodynamic occlusion of experimental choroidal neovascularisation and in a pilot study of chronic central serous chorioretinopathy [237]. This evidence belongs to pharmaceutical photodynamic therapy; direct nutritional ocular evidence for chlorophyll remains unavailable.

#### 4.6.5. Triterpenoids

Triterpenoids are lipophilic plant secondary metabolites with reported antioxidant, anti-inflammatory, and anti-angiogenic activities [238,239]. Ursolic acid, an isolated pentacyclic triterpenoid, improved corneal epithelial responses under hyperosmotic stress and reduced ocular–surface, lacrimal-gland, and meibomian-gland abnormalities in dry-eye models [240]. In oxygen-induced retinopathy models, it also reduced pathological neovascularisation and supported physiological vascular remodelling [241].

The constituents reviewed in this section provide different levels of evidence. DHA and other fatty acids have established structural roles and substantial mechanistic evidence, but clinical effects vary by disease, formulation, and route. Vitamins and carotenoids have nutrient- or formulation-specific evidence, while phenolic compounds, phytosterols, CoQ10, and triterpenoids are supported mainly by cellular, animal, or indirect evidence. Phospholipids and chlorophyll-derived compounds may function primarily as delivery or pharmaceutical materials in some studies. These distinctions provide the interpretative basis for the source-specific and application sections that follow.

## 5. Oil-Specific Evaluation of Natural Oils and Fats from Different Sources

Although individual minor constituents provide important mechanistic clues, natural oils and fats should not be interpreted simply as carriers of isolated bioactive molecules. Rather, each oil represents a source-specific lipid matrix in which major fatty acids, polar lipids, unsaponifiable compounds, and trace bioactive constituents coexist and interact. The ocular relevance of a given oil is therefore shaped not only by the presence of omega-3 or omega-6 PUFAs, carotenoids, tocopherols, phytosterols, or phenolic compounds, but also by its botanical or animal origin, tissue source, extraction method, degree of refining, oxidative stability, route of administration, and formulation context. This matrix-dependent perspective is particularly important for ocular diseases, in which nutritional support, tear-film lipid supplementation, epithelial barrier protection, anti-inflammatory activity, and retinal neuroprotection may require different lipid compositions and delivery strategies. Accordingly, the following section moves from component-based discussion to oil-specific evaluation. Natural oils and fats are grouped according to their major sources, including plant seed oils, fruit-derived oils, animal-derived lipids, and marine-derived oils, with emphasis on their compositional features, experimental evidence, and potential ocular applications.

### 5.1. Plant-Derived Oils

Plant oils are lipid-rich extracts obtained mainly from seeds and fleshy fruits. Their fatty-acid profiles, minor constituents, processing characteristics, and oxidative stability vary substantially according to botanical source. This section discusses seed oils followed by fruit-derived oils, focusing on their composition and evidence relevant to ocular health.

#### 5.1.1. Seed Oil

Seed oils are obtained from lipid-storage tissues, including seeds, endosperm, cotyledons, embryos, and bran or germ fractions through mechanical pressing, solvent extraction, or emerging green-extraction technologies [242]. Unlike olive oil and palm oil, which are derived mainly from fruit pulp, seed oils originate from a broad range of oil-bearing plant species [243]. Representative examples include rapeseed, soybean, sesame, perilla, safflower, sunflower, flaxseed, castor, borage, pumpkin-seed, corn, rice-bran, wheat-germ, and pine-nut oils [244].

Oil content, fatty-acid composition, and minor constituents vary substantially among seed oils [245] (Table 2). These differences influence their nutritional characteristics, oxidative stability, bioavailability, safety, and potential ocular applications [246].

**Table 2:** Fatty-acid composition of representative terrestrial plant oils discussed in relation to ocular health.

Oil	Main Fatty-Acid Feature	LA Range	GLA Range	ALA Range	Unit	Ref.
Castor oil	Ricinoleic-acid dominant; PUFA is not the main feature	1.33–5.48		0.13–0.51	% total fatty acids	[247]
Soybean oil	LA-rich oil with appreciable ALA	48.0–59.0		4.5–11.0	% total fatty acids	[248]
Perilla oil	ALA-rich plant oil	15.4		62.6	g/100 g	[249,250]
Sesame oil	LA-dominant oil with very low ALA	36.9–47.9		0.2–1.0	% total fatty acids	[248]
Safflower oil	High-LA omega-6 plant oil	67.8–83.2		ND–0.1	% total fatty acids	[248]
Flaxseed/linseed oil	ALA-rich plant oil	8.3–30.0		43.8–70.0	% total fatty acids	[248]
Evening primrose oil	Functional omega-6 oil characterized by LA and GLA	60.80–70.59	8.34–11.5	ND	% total fatty acids	[247]
Rapeseed/canola oil	MUFA-dominant oil containing LA and ALA	15.0–30.0		5.0–14.0	% total fatty acids	[248]
Almond oil	Oleic-acid dominant, followed by LA; very low ALA	20.0–30.0		ND–0.5	% total fatty acids	[248]
Walnut oil	High LA with appreciable ALA	54.0–65.0		9.0–15.4	% total fatty acids	[248]
Hemp seed oil	Relatively balanced LA/ALA with minor GLA	51.03–59.63	0.25–0.78	15.7–19.4	% total fatty acids	[247]
Borage seed oil	GLA-rich functional omega-6 oil	35.78–37.31	22.77–24.27		% total fatty acids	[251]
Olive oil	Oleic-acid/MUFA dominant; low PUFA	2.5–21.0		≤1.0	% total fatty acids	[252]
Coconut oil	Saturated/lauric-acid dominant; low PUFA	1.0–2.5		ND–0.2	% total fatty acids	[248]
Sea buckthorn berry oil	Berry/pulp oil is characterized by palmitoleic and palmitic acids, with some ALA	5.99–8.41		1.06–2.48	% total fatty acids	[247]
Avocado oil	Oleic-acid dominant; LA is the main minor PUFA	7.05–14.45		ND	% total fatty acids	[247]
Palm oil	Palmitic and oleic acids dominant; LA low and ALA trace	9.0–12.0		ND–0.5	% total fatty acids	[248]

Notes: Values are presented as ranges reported in the cited sources. LA, linoleic acid; GLA, γ-linolenic acid; ALA, α-linolenic acid; MUFA, monounsaturated fatty acid; PUFA, polyunsaturated fatty acid; ND, not detected or below the detection limit. Blank cells indicate that the corresponding value was not reported in the cited source or was not applicable. Values expressed as g/100 g and percentages of total fatty acids are reported according to their original analytical bases and should not be directly compared.

##### Castor Oil

Castor oil is obtained from the seeds of *Ricinus communis L.* and is used mainly in pharmaceutical, cosmetic, and topical lipid formulations rather than as a conventional cooking oil [253,254,255]. Its fatty-acid profile is characterised by approximately 86–92% ricinoleic acid, together with smaller amounts of oleic acid, LA, palmitic acid, and stearic acid [255,256] (Table 2). The hydroxyl group of ricinoleic acid contributes to the emulsifying, lubricating, film-forming, and surface-active properties of castor oil, supporting its use in ocular–surface lipid supplementation and ophthalmic emulsions [257,258].

Ophthalmic research on castor oil has focused mainly on MGD, evaporative DED, and blepharitis. Meibomian lipids reduce tear evaporation, facilitate lipid-layer spreading, and maintain tear-film stability. Disruption of their composition or secretion can therefore contribute to tear hyperosmolarity, ocular-surface injury, and dry-eye symptoms [259]. Castor oil-containing topical formulations have been investigated principally as supplements to this deficient or unstable tear-film lipid layer.

Early randomised, double-blind, placebo-controlled crossover evidence in patients with non-inflamed obstructive MGD showed that low-concentration homogenised eye drops containing 2% castor oil significantly improved symptom scores, tear interferometry grades, tear evaporation, rose bengal staining, tear breakup time, and obstruction scores of meibomian-gland orifices. Tear breakup time increased from 4.6 ± 2.8 s during the placebo period to 12.0 ± 3.5 s during the castor oil period, with no treatment-related complications observed. These findings suggest that castor oil may improve lipid-deficient dry eye by enhancing tear-film lipid-layer stability [129] (Table 1).

An open-label pilot study subsequently showed that, following a single instillation of a castor oil-containing ophthalmic emulsion, castor oil remained detectable in tears for up to 4 h. This was accompanied by short-term increases in tear lipid levels and lipid-layer thickness and reductions in dryness, itching, burning, and foreign-body sensation [130] (Table 1).

Real-world studies of Optive Plus^® ^(Allergan, Inc., Irvine, CA, USA), a multi-ingredient artificial tear formulation containing castor oil, also reported improvements in dry-eye signs and symptoms with generally favourable tolerability; these findings apply to the complete formulation [133] (Table 1). Evidence involving whole castor oil is more limited. In a randomised paired-eye study, periocular application of 100% preservative-free cold-pressed castor oil improved several signs of anterior blepharitis and MGD, including lid-margin thickening, telangiectasia, eyelash crusting, cylindrical dandruff, and lid-wiper epitheliopathy [125] (Table 1). Importantly, the oil was applied to the eyelids rather than instilled directly into the eye.

A review of the available evidence concluded that castor oil-containing preparations may increase tear-film lipid-layer thickness, prolong ocular-surface residence, improve tear-film stability, and alleviate symptoms associated with DED, MGD, and blepharitis. However, substantial heterogeneity was identified in formulation composition, dosing frequency, study design, and follow-up duration [260].

The safety of castor oil depends on product quality, concentration, route of administration, sterility, and formulation. The preparations evaluated in ophthalmic studies were low-concentration homogenised eye drops, formulated emulsions, or preservative-free products intended for periocular application and are not equivalent to industrial-grade, cosmetic-grade, or non-sterile castor oil [261]. Cosmetic safety assessments of castor oil, ricinoleates, and ricinoleic-acid esters under specified use conditions do not independently establish the safety of direct ocular or intraocular administration [262].

Overall, castor oil-related clinical evidence is concentrated in ocular-surface disorders. Eye-drop studies mainly evaluate castor oil-containing ophthalmic formulations, whereas the available whole-oil evidence concerns periocular eyelid application. Current findings provide preliminary support for lipid-layer supplementation in DED, MGD, and blepharitis but do not establish efficacy in retinal, lenticular, glaucomatous, or other intraocular diseases. Larger controlled trials using standardised formulations and longer follow-up are needed to define optimal concentrations, dosing schedules, target populations, and long-term ocular safety.

##### Flaxseed Oil and Evening Primrose Oil

Among plant seed oils, flaxseed oil and evening primrose oil (EPO) have been evaluated in human studies of dry eye and ocular–surface discomfort. Their evidence is considered separately below because the available studies include both oral whole oil interventions and multi-ingredient ophthalmic formulations, which support different levels of inference [263,264].

##### Flaxseed Oil

Flaxseed oil, also known as linseed oil, is obtained from the seeds of *Linum usitatissimum* L. and is characterised by a high proportion of ALA. Depending on cultivar and geographical origin, ALA accounts for approximately 55.7–60.1% of total fatty acids, followed by oleic acid (15.9–20.3%), LA (13.3–16.6%), palmitic acid (4.9–8.1%), and stearic acid (3.3–4.5%) [265,266] (Table 2). Flaxseed oil also contains smaller amounts of tocopherols, phytosterols, phenolic compounds, carotenoids, and chlorophyll. These minor constituents contribute to its compositional characteristics and oxidative stability [267].

Lignans such as secoisolariciresinol diglucoside are concentrated mainly in the seed coat, whole seed, and press cake and are generally less abundant in the oil fraction [268]. Accordingly, studies of whole flaxseed, flaxseed meal, or flaxseed-fortified foods [269] represent different dietary exposures from flaxseed oil. The high PUFA content of flaxseed oil also makes it susceptible to oxidation during heating and storage, which may affect its composition and biological activity [266]. An older preclinical study compared rats fed diets containing 10% hydrogenated coconut oil, safflower oil, or linseed (flaxseed) oil under chronic and acute light-stress conditions. Retinal susceptibility depended on both dietary-fat composition and light history, and the n-3-rich linseed-oil diet was associated with greater photoreceptor loss than the safflower oil or hydrogenated coconut oil diets under selected experimental conditions [270] (Table 3). This context-dependent finding does not establish that flaxseed oil is generally harmful to the retina, but it cautions against assuming that a high n-3 fatty-acid content is uniformly retinoprotective.

**Table 3:** Animal in vivo evidence.

Ocular Model	Tested Material (Category)	Species/Model (*n*)	Comparator	Dose/Route/Duration	Ocular Endpoint(s)	Main Finding	Permitted Interpretation	Ref.
Chronic/acute retinal light stress	Hydrogenated coconut, safflower or flaxseed oil diets; whole-oil comparison	Albino rats	Vehicle/model control	10% (*w*/*w*) oil diets; continued for 9 weeks after weaning; chronic or acute light stress	Photoreceptor loss; light-damage susceptibility; fatty-acid effects	Dietary fatty acids altered the light-stress response; an omega-3-rich diet increased photoreceptor damage versus omega-6-rich and omega-3-deficient diets	Preclinical; context-dependent; the omega-3-rich diet increased susceptibility to light damage	[270]
Selenite-induced cataract	Sesamol; isolated constituent	21 Sprague-Dawley rat pups; 7/group	Vehicle/model control	Selenite SC, postnatal day 9; sesamol 50 mg/kg/day IP, days 10–14	Lens opacity; MDA; TOS; GSH; TAS	Reduced lens opacity, MDA and TOS; increased GSH and TAS	Preclinical constituent evidence; not whole oil	[271]
Immune-mediated dry eye	Olive-pomace polyphenol extract + hydroxytyrosol; oil-derived phenolics	Desiccating-stress mouse DED model	Vehicle/model control	Topical hydroxytyrosol 100 µM or extract 0.20 mg/mL; 14 days	Corneal staining; lymph-node immune cells; inflammatory-gene expression	Improved corneal staining; modulated immune/inflammatory markers	Preclinical extract/compound evidence; not whole olive oil	[272]
Hypertension-induced retinal oxidative stress	Acebuche (wild olive) oil-enriched diet; whole natural oil	Hypertensive rodent retinal-injury model	Hypertensive control diet; conventional EVOO	12% (*w*/*w*) Acebuche oil-enriched diet; 6 weeks	Retinal redox status; NADPH-oxidase pathways; structural injury	Improved oxidative-stress and injury measures; greater effects than EVOO for selected outcomes	Supportive preclinical whole-oil evidence	[273]
Hypertensive retinal oxidative/inflammatory injury	Acebuche (wild olive) oil-enriched diet; whole natural oil	L-NAME-induced hypertensive mouse model	Vehicle/model control	12% (*w*/*w*) Acebuche-oil-enriched diet; 6 weeks	Oxidative stress; PPARγ/PPARα; IL-10; IL-1β/IL-6/TNF-α; COX-2; tissue damage	Reduced oxidative stress and pro-inflammatory markers; increased PPARγ, PPARα and IL-10	Supportive preclinical whole-oil evidence	[274]
High-tension glaucoma/ocular hypertension	Acebuche (wild olive) oil-enriched diet; whole natural oil	24 male C57BL/6 mice; MCE-induced glaucoma	Vehicle/model control	12% (*w*/*w*) Acebuche oil diet; 6 weeks; 2% MCE, 5 µL intracameral	IOP; glial activation; inflammatory/oxidative markers; apoptosis; ERG/PERG; RGC function	No IOP reduction; reduced glial activation, inflammation, oxidative stress, ischaemia and apoptosis; preserved RGC/visual function	Supportive preclinical whole-oil evidence	[275]
Ocular rewetting safety	Virgin coconut oil eye drops; whole natural oil	9 female albino New Zealand rabbits	Vehicle/model control	Topical VCO three times daily for 2 weeks; volume per instillation: Not reported	NIBUT; anterior-eye assessment; staining; pH; Schirmer test	Broadly comparable short-term measures; transient eyelid inflammation/slight irritation	Short-term rabbit tolerability; preliminary evidence	[276]
Light-induced retinal degeneration	Oral coconut oil; whole natural oil	Male Wistar rats	Vehicle/model control	5 or 10 mL/kg/day; 14 days pre- + 7 days post-light exposure; 5000 lux, 2 h	Retinal MDA; caspase-3; antioxidant capacity; morphology	Reduced MDA and caspase-3; better-preserved retinal morphology	Supportive preclinical whole-oil evidence	[277]
Dry eye/reduced tear secretion	Sea buckthorn pulp/seed oils + palmitoleic acid; whole oils + isolated fatty acid	Mouse dry-eye model	Vehicle/model control	Pulp oil 2.5 mL/kg/day or palmitoleic acid 0.75 mg/kg/day orally; 10 days	Tear secretion; lacrimal inflammatory cytokines; palmitoleate effects	Pulp oil, not seed oil, restored tears; palmitoleate preserved tears and reduced cytokines	Preclinical; pulp oil, seed oil and palmitoleate interpreted separately	[278]
Retinoid metabolism/visual cycle	Refined red palm-pressed mesocarp olein; whole-oil fraction	Rats	Vehicle/model control	Oral PPMO 2 g/kg/day; 84 days	RBP4; CRBP1/LRAT; RPE65/RDH5; retinol; retinyl esters	Increased RBP4, CRBP1/LRAT, RPE65/RDH5, retinol and retinyl esters	Preclinical biochemical evidence; ocular relevance uncertain	[279]
MNU-induced retinal degeneration/photoreceptor apoptosis	DHA-rich and EPA/DHA diets; concentrated/isolated fatty acids	50-day-old female Sprague–Dawley rats	Vehicle/model control	MNU 50 mg/kg IP; 9.5% DHA or EPA/DHA diets; follow-up to 20 weeks	Retinal histology; damage incidence/severity; serum fatty acids	9.5% DHA prevented retinal degeneration; 4.75% DHA less effective; 10% LA worsened damage	Supportive preclinical DHA-rich-diet evidence; not whole oil	[280]
Stargardt disease/inherited macular degeneration	EPA/DHA omega-3 preparation; concentrated fatty acids	8-month-old ABCA4−/− mice + wild-type controls	Vehicle/model control	EPA 172 mg + DHA 34 mg/day by gavage; 3 months; AA/EPA target ~1–1.5	AA/EPA ratio; retinal fatty acids; photoreceptors; lipofuscin/A2E; C3	Photoreceptor protection; reduced lipofuscin-related and inflammatory/complement markers	Supportive preclinical fatty-acid evidence; no human Stargardt efficacy data	[281]
Hereditary glaucoma	EPA/DHA omega-3, ± timolol; concentrated fatty acids	DBA/2J glaucoma mice	Vehicle/model control	Fish oil (~270 mg/day by gavage) ± topical timolol 0.5% once daily; 3 months; dose adjusted to an AA/EPA ratio of 1–1.5	RGC survival; neuroinflammation; glaucomatous neurodegeneration	Protected retinal neurons/RGCs; reduced inflammatory response	Supportive preclinical fatty-acid evidence; no human glaucoma efficacy data	[282]

Notes: All findings are preclinical. Whole oils, isolated oil-associated constituents, and engineered formulations are distinguished according to the intervention actually tested. Animal findings should not be interpreted as evidence of clinical efficacy. “Not reported” indicates that the information was not specified in the original publication. Abbreviations: A2E, N-retinylidene-N-retinylethanolamine; AA, arachidonic acid; ABCA4, ATP-binding cassette subfamily A member 4; C3, complement component 3; COX-2, cyclooxygenase-2; CRBP1, cellular retinol-binding protein 1; DED, dry eye disease; DHA, docosahexaenoic acid; EPA, eicosapentaenoic acid; ERG, electroretinography; EVOO, extra-virgin olive oil; GSH, glutathione; IL, interleukin; IOP, intraocular pressure; IP, intraperitoneal; LA, linoleic acid; LRAT, lecithin retinol acyltransferase; MCE, methylcellulose; MDA, malondialdehyde; MNU, N-methyl-N-nitrosourea; NADPH, nicotinamide adenine dinucleotide phosphate; NIBUT, non-invasive tear breakup time; PERG, pattern electroretinogram; PPAR, peroxisome proliferator-activated receptor; PPMO, refined red palm-pressed mesocarp olein; RBP4, retinol-binding protein 4; RDH5, retinol dehydrogenase 5; RGC, retinal ganglion cell; RPE65, retinal pigment epithelium-specific 65-kDa protein; SC, subcutaneous; TAS, total antioxidant status; TNF-α, tumour necrosis factor-α; TOS, total oxidant status; VCO, virgin coconut oil.

Direct human evidence for flaxseed oil is limited primarily to ocular–surface disease [283,284]. In a randomised clinical trial involving women with keratoconjunctivitis sicca associated with Sjögren’s syndrome, oral flaxseed oil at 1 or 2 g/day for 180 days improved dry-eye symptoms, ocular–surface inflammation, Schirmer I test results, and tear breakup time compared with placebo [124] (Table 1). However, the study was based on a relatively small and disease-specific population, limiting the precision and generalisability of its findings.

Flaxseed oil has also been evaluated as a component of ophthalmic formulations. In a multicentre, double-blind randomised trial involving 242 participants with DED, the OM3 nanoemulsion—containing carboxymethylcellulose, glycerin, flaxseed oil, castor oil, trehalose, and other osmoprotective ingredients—was compared with a control artificial tear. Both groups showed improvements in the Ocular Surface Disease Index, ocular–surface staining, and tear breakup time, while OM3 showed advantages in selected staining outcomes [118] (Table 1). This trial provides evidence for the complete OM3 formulation rather than for flaxseed oil as an independently evaluated intervention.

Overall, the direct ocular evidence for whole flaxseed oil is based mainly on one small oral trial in Sjögren’s-syndrome-associated dry eye. Its additional clinical use in OM3 represents formulation-level evidence. These findings provide preliminary support for oral flaxseed oil and flaxseed oil-containing topical formulations in selected ocular–surface conditions, but the independent effect, optimal dose, target population, and long-term safety of flaxseed oil remain uncertain.

##### Evening Primrose Oil

Evening primrose oil is obtained from the seeds of *Oenothera biennis* L. and is commonly used as an oral nutritional supplement [285]. It is rich in omega-6 PUFAs, with LA accounting for approximately 70–74% and GLA for approximately 8–10% of total fatty acids [286] (Table 2). Its relatively high GLA content distinguishes EPO from most conventional LA-rich seed oils and provides the rationale for its investigation in inflammatory and ocular-surface conditions.

Direct ocular evidence is limited to oral supplementation. In a randomised, double-blind, placebo-controlled trial, 76 female soft-contact-lens wearers received EPO capsules or an olive oil placebo for 6 months. EPO improved self-reported dry-eye symptoms at 3 and 6 months, increased tear-meniscus height, and produced an approximately 20% improvement in contact-lens comfort from baseline. However, tear-film stability, meibomian-gland secretion, lipid-layer appearance, ocular–surface hyperaemia, and staining did not improve significantly [121] (Table 1).

These findings suggest a possible symptomatic benefit of oral EPO in female contact-lens wearers, but the limited changes in objective ocular–surface outcomes and the narrowly defined study population preclude broader conclusions for DED. Additional trials are required to determine whether the observed benefit extends to other dry-eye subtypes and to establish the relevant dose, duration, and long-term safety.

Overall, flaxseed oil and EPO have limited but relatively direct human evidence in ocular–surface conditions. Flaxseed oil has been evaluated as an oral whole oil intervention and as one component of a multi-ingredient artificial tear, whereas EPO evidence is restricted to oral supplementation in contact lens-associated ocular discomfort. Neither oil currently has sufficient evidence to be considered an established treatment for DED nor other ocular diseases.

#### 5.1.2. Fruit-Derived Oils

Fruit-derived oils are obtained mainly from lipid-rich fruit tissues, including the pulp, mesocarp, peel, fruit paste, or whole fruit, through mechanical pressing, centrifugation, solvent extraction, or other extraction technologies [287].

Their lipid content and composition vary according to plant species, tissue of origin, fruit maturity, extraction method, and postharvest processing [288] (Table 2).

In addition to TAGs, some fruit-derived oils retain variable amounts of carotenoids, phenolic compounds, phytosterols, tocopherols, pigments, and other constituents in their unsaponifiable fractions [289,290]. Oils recovered from fruit pulp, peel, pomace, or other processing fractions may consequently differ in composition and stability [291,292]. These source- and processing-related differences influence their nutritional characteristics, oxidative stability, bioavailability, safety, and potential ocular applications. The following subsections therefore consider individual fruit-derived oils separately, with emphasis on their composition and available ocular evidence.

##### Olive Oil

Olive oil is obtained from the fruit of *Olea europaea* L. and is a major dietary lipid source within Mediterranean dietary patterns [293,294,295]. Virgin olive oil and extra virgin olive oil (EVOO) are produced primarily by mechanical processes and generally retain more phenolic and volatile constituents than refined olive oil [296,297]. Oleic acid is the predominant fatty acid, accompanied by smaller proportions of palmitic acid, LA, stearic acid, and ALA; their relative abundance varies with cultivar, geographical origin, fruit maturity, and processing [298,299,300] (Table 2). Minor constituents include hydroxytyrosol, tyrosol, oleuropein, oleocanthal, oleacein, squalene, tocopherols, and phytosterols [301]. Although higher olive oil intake has been associated with favourable cardiovascular and mortality outcomes [302,303,304], these systemic associations provide only an indirect nutritional context for ocular research.

Human ocular evidence is currently limited to observational dietary data. In the population-based Alienor study, regular olive oil consumption was associated with a lower risk of late AMD but not early AMD. Comparable associations were not observed for omega-3-enriched oils, omega-6-enriched oils, mixed oils, butter, or margarine [141] (Table 1). Because olive oil intake formed part of the participants’ broader dietary exposure, residual confounding and other characteristics of the dietary pattern cannot be excluded. No randomised clinical trial has established that olive oil prevents AMD progression or treats an ocular disease.

Preclinical whole oil evidence has focused mainly on wild olive oil, commonly referred to as Acebuche oil. In hypertension-related retinal injury models, Acebuche oil-enriched diets improved selected retinal oxidative-stress parameters and showed greater effects than conventional EVOO for some outcomes [273] (Table 3). In related experimental models, Acebuche oil increased peroxisome proliferator-activated receptor-γ (PPARγ), PPARα, and interleukin-10 (IL-10) and reduced interleukin-1β (IL-1β), IL-6, tumour necrosis factor-α (TNF-α), and COX-2 expression [274] (Table 3). In mouse models of ocular hypertension-induced glaucoma, Acebuche oil-enriched diets also attenuated retinal glial activation, inflammation, oxidative stress, ischaemia, apoptosis, and electrophysiological dysfunction [275] (Table 3). These studies provide preclinical evidence for dietary Acebuche oil but have not yet been replicated in human ocular disease.

Olive-derived phenolic constituents and extracts have been investigated separately. In human corneal and conjunctival epithelial cell models, olive pomace phenolic extracts reduced reactive oxygen species generation and inflammatory-mediator release [305] (Table 4). In preclinical dry-eye models, olive pomace polyphenol extracts and hydroxytyrosol modulated inflammation-related gene expression and improved corneal fluorescein staining [272] (Table 3). These findings provide constituent- and extract-level mechanistic evidence relevant to ocular–surface inflammation.

Acebuche oil has additionally been incorporated into engineered ophthalmic nanoemulsions. These formulations showed particle size, osmolarity, pH, and colloidal stability compatible with ocular administration and reduced corneal and retinal injury and nicotinamide adenine dinucleotide phosphate oxidase-related oxidative responses in experimental hypertension models [306] (Table 5). These results support the pharmaceutical feasibility and preclinical performance of the tested nanoemulsion.

**Table 4:** Cell-based mechanistic evidence.

Cell/Context	Tested Material (Category)	Concentration/Exposure	Comparator/Stimulus	Endpoint(s)	Main Finding	Permitted Interpretation	Ref.
Developing rat retinal-cell cultures; photoreceptor apoptosis	DHA; isolated fatty acid	DHA 6.7 µM added after 1 day; developmental time course	DHA-free serum-free culture	Photoreceptor survival; apoptosis	Reduced selective photoreceptor apoptosis	Ocular-cell constituent evidence only	[307]
Human corneal epithelial cells and conjunctival epithelial cell models	Olive-pomace phenolic extracts, hydroxytyrosol and oleuropein; oil-derived phenolic extract/compounds	Extracts 0.005–0.80 mg/mL; hydroxytyrosol 1–150 µM; oleuropein 5–300 µM; pretreatment 1–2 h; TNF-α 25 ng/mL for 24 h or UVB 107.25 mJ/cm^2^	Vehicle/model control	IL-1β, IL-6, IL-8, IL-17A, IP-10 and ROS	Olive pomace extracts and hydroxytyrosol inhibited secretion of most measured interleukins in HCE cells; all samples reduced IP-10 in conjunctival epithelial cells and inhibited UVB-related ROS generation	Cell evidence only; not whole oil	[305]
Human peripheral CD4+ T cells (indirect, non-ocular cell evidence)	Olive-pomace polyphenol extract and hydroxytyrosol; oil-derived phenolic extract/compound	48-h exposure to olive-pomace extract 0.10–0.40 mg/mL or hydroxytyrosol 25–100 µM	vehicle/model control	CD4+ T-cell activation and inflammatory markers	The extract and hydroxytyrosol reduced selected T-cell activation and inflammatory measures	Preclinical extract/compound evidence; no direct evidence for whole olive oil	[272]
ARPE-19 retinal pigment epithelial cells	Vegetable oils + lutein/zeaxanthin; uptake vehicles	Lutein/zeaxanthin + 1% vegetable oil; 48 h	Vehicle/model control	Intracellular lutein/zeaxanthin by HPLC	Oil-dependent uptake; coconut oil highest, linseed oil lowest	Cell-uptake/vehicle evidence; no therapeutic efficacy	[308]

Notes: Direct ocular cell evidence and indirect non-ocular mechanistic evidence are identified separately. These studies support biological plausibility but do not establish clinical efficacy, and findings obtained with isolated constituents or extracts are not attributed to their source oils. Abbreviations: ARPE-19, human retinal pigment epithelial cell line; CD4, cluster of differentiation 4; DHA, docosahexaenoic acid; HCE, human corneal epithelial cells; HPLC, high-performance liquid chromatography; IL, interleukin; IP-10, interferon-γ-induced protein 10; ROS, reactive oxygen species; TNF-α, tumour necrosis factor-α; UVB, ultraviolet B.

**Table 5:** Ophthalmic formulation and drug-delivery evidence.

Application	Tested System (Category)	Experimental Model(s)	Composition/Regimen	Delivery/Safety Endpoint(s)	Main Finding	Permitted Interpretation	Ref.
Ocular epalrestat delivery/diabetic-eye platform	Epalrestat-loaded soybean-oil oleogel/oleorod; engineered oleogel	Ocular delivery models; HET-CAM; ex vivo/in vivo evaluation	Epalrestat-loaded soybean-oil oleogel/oleorod; gelator 5% or 10% *w*/*w*; 24-h release; in ovo/ex vivo/in vivo	Drug release; irritation; ocular retention; delivery feasibility	10% gelator formed solid oleorods; ~4 μg epalrestat/mg released (~10%/24 h); no HET-CAM irritation	Preclinical formulation-performance evidence only	[309]
Ocular delivery vehicle development	Almond-oil nanoemulsion; engineered oil phase	Ophthalmic nanoemulsion formulations	Almond oil; HEPES/Palitzsch buffer; Tween 20/80; in vitro characterization	Size; PDI; zeta potential; microviscosity; stability; ocular suitability	Selected formulations: suitable size distribution, negative zeta potential, stable	Formulation-performance evidence only	[310]
Hypertension-related ocular oxidative stress/retinal injury	Acebuche-oil ophthalmic nanoemulsion; whole-oil formulation	Hypertension-related ocular oxidative-stress model	6% (*w/v*) Acebuche-oil nanoemulsion; one corneal drop every 8 h for 5 days, or one or two 1-μL intravitreal injections	Oxidative stress; retinoprotection; formulation performance; tolerability	Reduced hypertension-induced ocular oxidative injury; oculoprotective effects	Preclinical formulation evidence; oil/formulation roles unresolved	[306]
Ocular inflammation: delivery/efficacy comparison	Difluprednate 0.05% emulsion vs. 0.04% solution; pharmaceutical oil-phase formulation	Rabbit ocular PK and uveitis models	0.04% solution BID vs. 0.05% emulsion QID	Ocular PK; inflammation score; cell count; PGE2; protein; tolerability	Comparable anti-inflammatory efficacy between solution and emulsion	Product-comparison evidence; oil-phase contribution not isolated	[311]
Posterior-segment fluocinolone-acetonide delivery	Cow-ghee-fortified fluocinolone-acetonide microemulsion; lipid excipient	In vitro; ex vivo permeation/irritation; rat PK	Lauroglycol/Labrasol/Transcutol microemulsion + cow ghee	Permeation; irritation; posterior retention; PK	Cow ghee increased permeation and posterior retention; irritation outcome not reported	Formulation/permeation evidence only; no intrinsic cow-ghee efficacy	[312]
Topical posterior-segment fingolimod delivery	Fingolimod-loaded soybean-oil nanoemulsion; engineered oil phase	Preformulation; in vitro characterization; ocular-delivery evaluation	Oil screening; optimized fingolimod-loaded soybean-oil nanoemulsion	Solubility; size; PDI; zeta potential; pH; stability; release; delivery	Soybean/castor oils: higher fingolimod solubility; optimized soybean formulation met delivery criteria	Engineered-delivery evidence; no intrinsic soybean-oil efficacy	[313]
Ocular mangiferin delivery	Mangiferin-loaded NLCs; engineered lipid-delivery system	ARPE-19 assays; ex vivo bovine-corneal permeation	Mangiferin-loaded/blank NLCs; H_2_O_2_ challenge	Cytocompatibility; ROS; glutathione; permeation; antioxidant activity	ARPE-19 compatible; preserved antioxidant activity; supported corneal permeation	In vitro/ex vivo formulation evidence; clinical efficacy untested	[314]
Sustained glaucoma drug delivery	Latanoprost-loaded EggPC liposomes; phospholipid-delivery system	In vitro release; rabbit subconjunctival administration	Single subconjunctival injection vs. daily topical latanoprost; 90 days	Size; loading; stability; release; IOP; ocular inflammation	Gradual release; IOP reduction maintained up to 90 days; no visible inflammation	Preclinical engineered-liposome evidence; not egg-yolk-oil efficacy	[315]

Notes: Evidence is classified according to the formulation or delivery system actually tested. Improvements in drug solubility, stability, release, retention, permeation, pharmacokinetics, or ocular response are interpreted at the formulation level and are not attributed to an intrinsic therapeutic effect of the carrier oil or lipid excipient. Reported safety or tolerability applies only to the tested formulation, concentration, route, and model. All findings are preclinical and do not establish clinical efficacy. Abbreviations: ARPE-19, human retinal pigment epithelial cell line; BID, twice daily; EggPC, egg-derived phosphatidylcholine; HEPES, 4-(2-hydroxyethyl)-1-piperazineethanesulfonic acid; HET-CAM, hen’s egg test–chorioallantoic membrane; H_2_O_2_, hydrogen peroxide; IOP, intraocular pressure; NLC, nanostructured lipid carrier; PDI, polydispersity index; PGE2, prostaglandin E2; QID, four times daily; PK, pharmacokinetics; ROS, reactive oxygen species.

Overall, evidence concerning olive oil and ocular health remains heterogeneous. Human evidence is restricted to an observational association between dietary olive oil consumption and late AMD. Dietary Acebuche oil has shown retinal effects in animal models, olive-derived phenolic compounds, and extracts provide predominantly preclinical mechanistic evidence, and Acebuche oil nanoemulsions provide formulation-level evidence. These findings do not yet establish the clinical efficacy of olive oil for AMD, DED, glaucoma, or other ocular diseases. Future studies should evaluate well-characterised oils in controlled human populations, with documentation of olive variety, processing method, fatty-acid and phenolic composition, dose, route of administration, oxidative status, and long-term safety.

##### Coconut Oil

Coconut oil is obtained from the kernel of *Cocos nucifera* L. and is widely used as an edible oil in tropical and subtropical regions [316]. Conventional coconut oil is commonly produced from dried coconut kernel, whereas virgin coconut oil (VCO) is obtained from fresh mature kernel or coconut milk using physical extraction methods without conventional chemical refining, bleaching, or deodorisation [317,318]. These differences in raw material and processing can affect the retention of phenolic compounds and other minor constituents.

Coconut oil differs from most unsaturated fatty-acid-rich plant oils because it contains a high proportion of saturated fatty acids. Lauric acid is the predominant fatty acid, accounting for approximately 47.68–50.08% in cold- and hot-pressed oils, together with caprylic, capric, myristic, palmitic, stearic, oleic, and linoleic acids [319] (Table 2). Meta-analyses indicate that coconut oil consumption may increase both high-density lipoprotein cholesterol and low-density lipoprotein cholesterol compared with non-tropical plant oils [320,321,322]. These systemic findings are relevant to the dietary safety context but do not provide evidence of ocular benefit.

Lauric acid and its glycerol ester monolaurin have demonstrated antimicrobial and immunomodulatory activities in experimental systems [323,324]. VCO also contains small amounts of phenolic compounds, tocopherols, and phytosterols that may contribute to its experimentally observed antioxidant and anti-inflammatory properties [325]. These findings concern individual constituents or predominantly non-ocular experimental models.

Human ocular evidence for coconut oil remains sparse. Coconut oil-containing ingredients have also been used in multi-ingredient eyelid-cleansing products investigated for blepharitis and Demodex infestation [326]. The reported findings apply to the complete cleansing formulation rather than to coconut oil alone.

Animal studies provide limited information on topical tolerability and retinal effects. In an open-label pilot study involving nine female New Zealand White rabbits, VCO was compared with Tears Naturale II and saline as an ocular rewetting agent. Assessments showed no significant changes in the measured tear-film or anterior-segment parameters, although transient eyelid inflammation with slight irritation was observed [276] (Table 3).

In a rat model of light-induced retinal degeneration, animals received coconut oil at 5 or 10 mL/kg for 14 days before exposure to 5000 lux white light for 2 h and for a further 7 days after exposure. Coconut oil reduced retinal malondialdehyde levels and caspase-3 activity and preserved retinal histological structure, with more apparent effects at 10 mL/kg [277] (Table 3). A coconut oil-based delivery matrix produced greater cellular uptake of lutein and zeaxanthin than the other tested plant oil matrices in ARPE-19 cells [308] (Table 4).

Overall, coconut oil-related ocular evidence is limited and heterogeneous. Human observations concern short-term ocular–surface applications and multi-ingredient eyelid-cleansing products; rabbit data address topical tolerability; rat studies provide preclinical whole-oil evidence in light-induced retinal injury; and ARPE-19 experiments concern carotenoid delivery. No controlled human evidence currently supports oral coconut oil for the prevention or treatment of ocular disease. Further studies should use well-characterised, sterile formulations and clearly define coconut oil type, concentration, route of administration, comparator, ocular outcomes, and longer-term safety.

##### Sea Buckthorn Oil

Sea buckthorn (*Hippophae rhamnoides* L.) produces compositionally distinct oils from its pulp and seeds. Although sea buckthorn seed oil is botanically a seed oil, the two oils are considered together here because they are frequently marketed under the general term “sea buckthorn oil” (SBO) and have been directly compared in ocular studies. Sea buckthorn pulp oil is characterised by relatively high levels of palmitoleic acid and carotenoids, whereas seed oil contains higher proportions of LA, ALA, and phytosterols [327,328]. The source tissue should therefore be specified when interpreting SBO-related evidence.

Human evidence is concentrated in oral supplementation studies of dry-eye symptoms. In a double-blind, randomised, placebo-controlled trial involving adults aged 20–75 years with dry-eye symptoms, oral administration of 2 g/day SBO for 3 months attenuated the seasonal increase in tear-film osmolarity during the cold season and improved selected symptoms, including ocular redness and burning [120] (Table 1). This study provides controlled evidence for the tested oral SBO product, although the benefit was modest and not consistently demonstrated across all ocular–surface outcomes.

In a subsequent randomised controlled study, 12 weeks of oral SBO soft-gel supplementation improved self-reported ocular dryness, foreign-body sensation, redness, and ocular moisturising sensation. Supplementation was also associated with increased serum catalase activity and reduced tumour necrosis factor-α levels [138] (Table 1). However, the principal ocular outcomes were questionnaire-based, with limited objective assessment of tear-film or ocular–surface function.

SBO has also been incorporated into a closed-eye eyelid spray containing sodium hyaluronate. The formulation reduced Ocular Surface Disease Index scores and improved subjective dryness and tearing [137] (Table 1). Because both SBO and sodium hyaluronate were components of the tested spray, this study provides evidence for the complete ocular–surface formulation.

Source-specific differences were demonstrated more clearly in an experimental mouse model of dry eye. Oral sea buckthorn pulp oil restored aqueous tear secretion and suppressed inflammation-related cytokine expression in the lacrimal gland, whereas seed oil did not produce a comparable effect. Isolated palmitoleate, which is enriched in pulp oil, showed similar effects on tear secretion and lacrimal gland inflammation [278] (Table 3). These findings indicate that pulp and seed oils are not functionally interchangeable and provide constituent-level support for a possible contribution of palmitoleate to the activity observed with pulp oil.

Overall, SBO has limited but direct human evidence in dry eye-related oral supplementation, together with product-level evidence from an SBO-containing eyelid spray. The available trials were relatively short and relied partly on subjective outcomes, while oil source and composition were not always sufficiently characterised. Animal evidence further suggests that pulp oil and seed oil may differ in their effects on tear secretion. Future trials should distinguish pulp oil from seed oil, report their fatty-acid and carotenoid composition, and incorporate objective outcomes such as tear osmolarity, tear breakup time, Schirmer testing, ocular-surface staining, and inflammatory markers. Larger samples and longer follow-up are also needed to establish effective doses and long-term safety.

##### Avocado Oil

Avocado oil is obtained primarily from the mesocarp of *Persea americana* Mill. through mechanical pressing, aqueous extraction, solvent extraction, or other extraction technologies [329,330,331]. It is rich in monounsaturated fatty acids, particularly oleic acid, and it also contains palmitic acid, LA, palmitoleic acid, and small amounts of ALA. Its composition varies with cultivar, fruit maturity, geographical origin, processing tissue, and extraction method [288,289,329,331] (Table 2). Minor constituents include phytosterols, tocopherols, carotenoids, chlorophylls, and phenolic compounds [332,333].

Direct ocular research specifically evaluating extracted avocado oil remains scarce [334,335]. Its principal relevance to ocular nutrition is its potential to facilitate the absorption of lipophilic carotenoids. Lutein and zeaxanthin accumulate in the macula and contribute to short-wavelength light filtration and retinal antioxidant defence [64,336]. Human feeding studies have shown that adding avocado or avocado oil to carotenoid-containing foods, including salads and tomato sauce, can increase carotenoid absorption [337].

In a 2017 randomised controlled trial, daily consumption of one whole avocado for 6 months increased serum lutein concentrations and macular pigment density in older adults [142] (Table 1). This study is retained because avocado is a lipid-rich fruit in which the intrinsic oil fraction forms part of the dietary matrix that supports the delivery and absorption of lutein and other lipophilic constituents. It is therefore considered whole-food evidence relevant to the nutritional function of avocado-derived lipids rather than a direct avocado oil intervention.

Overall, current evidence supports a possible role for avocado-derived lipids in carotenoid bioavailability and macular pigment nutrition, but direct evidence for extracted avocado oil in ocular disease is absent. Studies using compositionally characterised avocado oil and defined ocular endpoints are required before conclusions can be drawn regarding its efficacy, dose, route of administration, or long-term safety.

##### Palm Oil

Palm oil is obtained from the mesocarp of the oil palm fruit (*Elaeis guineensis* Jacq.) and is distinct from palm kernel oil, which is extracted from the seed kernel. Red palm oil and minimally refined palm fruit oil fractions can retain relatively high concentrations of α-carotene, β-carotene, tocopherols, and tocotrienols [338,339,340]. Their composition differs from that of conventionally refined palm oil, palm kernel oil, and high-oleic palm oil, particularly in fatty-acid profile, carotenoid content, vitamin E homologues, and oxidative stability [199,341].

Direct ocular evidence for palm oil remains limited. Its nutritional relevance is associated mainly with provitamin A carotenoids and vitamin E-family compounds. α-Carotene and β-carotene can contribute to vitamin A status and retinaldehyde availability, while tocopherols and tocotrienols participate in antioxidant and inflammatory regulation [342,343]. Red palm oil may therefore have nutritional relevance to vitamin A deficiency-related ocular conditions, including night blindness and xerophthalmia.

Preclinical evidence has been reported for refined red palm-pressed mesocarp olein (PPMO), an intermediate palm fruit oil fraction. In rats receiving PPMO by daily gavage at 2 g/kg for 84 days, the intervention increased retinol and retinyl ester levels and upregulated proteins involved in retinol transport, esterification, and the visual cycle, including retinol-binding protein 4, cellular retinol-binding protein 1, lecithin retinol acyltransferase, retinal pigment epithelium-specific 65 kDa protein, and retinol dehydrogenase 5 [279] (Table 3). This study provides evidence that the tested PPMO fraction can influence systemic and ocular retinoid metabolism in an animal model.

Overall, palm oil-related ocular evidence is currently restricted to its nutritional composition and one preclinical study of a processed palm fruit oil fraction. No direct clinical evidence demonstrates that conventional palm oil, red palm oil, or PPMO prevents or treats ocular disease. Future studies should specify the palm oil fraction, refining status, carotenoid and tocotrienol content, dose, and route of administration and should evaluate clinically relevant ocular endpoints and safety.

### 5.2. Terrestrial Animal-Derived Fats and Lipid Materials

The terrestrial animal-derived materials considered in this review comprise two distinct categories: triacylglycerol-rich dietary fats and isolated or processed animal-derived lipids used as formulation materials. This distinction is important because compositional information on dietary animal fats does not constitute evidence of ocular efficacy, while the pharmaceutical function of an isolated lipid material cannot be attributed to the corresponding whole fat or source tissue.

Terrestrial animal-derived dietary fats include lard, poultry fat, beef and mutton tallow, and milk-derived fats such as butter. These fats consist predominantly of TAGs and have source-specific fatty-acid profiles, with varying amounts of cholesterol and other minor lipophilic constituents. Their composition and oxidative stability depend on animal species, tissue source, diet, rendering conditions, and subsequent processing (Table 6) [344,345,346]. Their inclusion in this review provides compositional context for comparing terrestrial animal fats with plant- and marine-derived lipid sources and should not be interpreted as evidence of an ocular-health benefit.

**Table 6:** Fatty-acid profiles of representative animal- and marine-derived lipid sources relevant to ocular health.

Lipid Sources	Main Lipid Characteristic	LA Range	GLA Range	ARA	ALA Range	EPA	DPA	DHA	Unit	Ref.
Egg yolk oil	LA-, ARA-, and DHA-containing animal lipid	134		17	4	0		4	g/kg total FA	[249]
Anchovy oil	EPA- and DHA-rich fish oil	ND–3.5	ND–5.0	1.25	ND–7.0	15.5	2	15.25	% total fatty acids	[347]
Tuna oil	DHA-rich fish oil	ND–3.0	ND–4.0	1.5	ND–2.0	5.75	1.5	31.75	% total fatty acids	[347]
Krill oil	Phospholipid-rich marine omega-3 lipid	ND–3.0			0.1–4.7	21.15	0.35	11.4	% total fatty acids	[347]
Menhaden oil	Named EPA/DHA-rich fish oil	2.0–3.5	ND–2.5	1	ND–2.0	15.75	2.5	8.25	% total fatty acids	[347]
Salmon oil (wild)	Wild salmon oil	1.5–2.5	ND–2.0	1.5	ND–2.0	9	2.25	10	% total fatty acids	[347]
Salmon oil (farmed)	Farmed salmon-source oil with higher LA and ALA	8.0–15.0	ND–0.5	0.6	3.0–6.0	4	1.75	6.5	% total fatty acids	[347]
Calanus oil	Wax ester-rich marine lipid	0.7–1.5	ND–0.9	0.35	1.1–3.5	13.8	0.65	9.75	% total fatty acids	[347]
Cod liver oil	EPA- and DHA-containing fish liver oil	0.5–3.0		0.75	ND–2.0	11.5	1.75	12	% total fatty acids	[347]
*Schizochytrium* sp. dried microalgal biomass	DHA-rich microalgal biomass	0.15		0.59	0.97	1.9		202.62	mg/g dried biomass	[249]

Notes: Values are presented as single values or ranges reported in the cited sources. LA, linoleic acid; GLA, γ-linolenic acid; ARA, arachidonic acid; ALA, α-linolenic acid; EPA, eicosapentaenoic acid; DPA, docosapentaenoic acid; DHA, docosahexaenoic acid; FA, fatty acids; ND, not detected or below the detection limit. Blank cells indicate that the corresponding fatty acid was not reported in the cited source or was not applicable to that lipid source. Because the data are reported on different analytical bases (percentage of total fatty acids, g/kg total fatty acids, and mg/g dried biomass), values should be interpreted within their original reporting basis and should not be directly compared across rows.

Direct ocular evidence for non-formulated terrestrial animal fats is currently absent or extremely limited. Moreover, food-grade or rendered animal fats are not suitable for direct ocular application because their composition, oxidation status, sterility, viscosity, and biocompatibility are not controlled according to ophthalmic requirements [348]. Accordingly, lard, poultry fat, tallow, and butter are not considered further as ocular interventions in this review.

#### Egg-Derived Phospholipids as Formulation Materials in Multi-Ingredient Ophthalmic Emulsions

Egg yolk contains phosphatidylcholine, phosphatidylethanolamine, cholesterol, carotenoids, and fat-soluble vitamins [349,350,351]. In ophthalmic formulations, egg-derived phospholipids are used principally as amphiphilic and emulsion-stabilising materials that facilitate lipid spreading and stabilise oil-in-water interfaces. This pharmaceutical role is distinct from the administration of whole egg yolk oil.

The available human evidence concerns Emustil® (SIFI S.p.A., Catania, Italy), an oil-in-water emulsion containing 7% soybean oil and 3% egg-derived natural phospholipids. In a 2012 comparative study of lipid-deficient evaporative DED, sodium hyaluronate, hydroxypropyl methylcellulose, and Emustil® all improved symptoms and evaporation-related outcomes, while Emustil® produced greater improvements in tear-film stability, tear osmolarity, and corneal staining [134] (Table 1).

In a low-humidity exposure study involving 12 healthy male participants, prophylactic administration of Emustil® increased tear-film lipid-layer thickness and improved non-invasive tear breakup time [135] (Table 1). A subsequent evaluation under dry-environment conditions reported improvements in selected tear-film parameters and subjective discomfort when Emustil® was used either before or after environmental exposure [136] (Table 1).

Collectively, these studies provide product-level evidence for Emustil® ophthalmic emulsion. The evidence is limited by small samples, short environmental-exposure models, and the inclusion of healthy participants in some studies. Larger trials in clinically defined DED populations are needed to determine the magnitude and durability of benefit.

### 5.3. Marine-Derived Oils

Marine-derived oils include fish body oil, krill oil, cod liver oil, and microalgal oil. They differ in biological source, EPA/DHA content, lipid form, accompanying nutrients, processing requirements, and oxidative stability [352,353] (Table 6). Unlike most plant oils, which provide mainly ALA, LA, or oleic acid, marine oils can supply preformed EPA and DHA; endogenous conversion of ALA to EPA and particularly DHA is limited [354,355].

DHA is highly enriched in photoreceptor outer-segment membranes and contributes to membrane organisation, phototransduction, retinal integrity, and lipid-mediator generation [146]. Marine oils and marine-derived EPA/DHA preparations have consequently been investigated in retinal development, infant visual maturation, DED, AMD, and retinal degeneration [356,357]. The tested materials nevertheless range from source-defined whole oils to concentrated EPA/DHA preparations and multi-ingredient nutritional formulations [358,359].

#### 5.3.1. Fish Oil and Fish Oil-Derived Epa/Dha Preparations

Fish oil is obtained from lipid-rich fish tissues or fish-processing fractions, with common sources including anchovy, sardine, mackerel, salmon, and tuna [347]. Its EPA/DHA ratio varies with species, anatomical source, processing, concentration, and product standardisation [360,361]. Tuna oil, for example, may have a DHA-dominant profile, with one analysis reporting approximately 24.56% DHA and 7.81% EPA [362] (Table 6). Fish oils may also contain variable amounts of phospholipids, cholesterol, fat-soluble vitamins, squalene, and carotenoids, depending on source, refining, and oxidative status [82].

DHA can comprise a large proportion of photoreceptor outer-segment phospholipid fatty acids, and retinal uptake varies with molecular carrier form [363]. DHA-derived neuroprotectin D1 has also been associated with anti-apoptotic and pro-survival responses in oxidatively stressed retinal pigment epithelial cells [364,365].

Most experimental retinal studies have evaluated DHA or defined EPA/DHA preparations rather than unmodified fish oil. DHA reduced photoreceptor apoptosis in cultured rat retinal cells [307] (Table 4). In rats with N-methyl-N-nitrosourea-induced retinal injury, a diet containing 9.5% DHA attenuated retinal degeneration [280] (Table 3). In ABCA4-knockout mice, EPA/DHA supplementation reduced A2E, lipofuscin, and complement component 3 when the blood arachidonic acid/EPA ratio decreased to approximately 1.0–1.5 [281] (Table 3). In DBA/2J mice, 3 months of EPA/DHA gavage increased retinal ganglion-cell density and reduced tumour necrosis factor-α and interleukin-18, with greater effects when combined with timolol [282] (Table 3).

##### Clinical Evidence in Dry Eye Disease

The strongest clinical evidence is provided by the multicentre DREAM trial, in which participants received a triglyceride-form concentrate providing EPA 2000 mg plus DHA 1000 mg daily or placebo. Symptoms and major objective signs improved in both groups, but the EPA/DHA preparation was not superior to placebo [115] (Table 1). This neutral result should anchor the interpretation of omega-3 supplementation in a general DED population.

Several smaller trials reported more favourable outcomes. EPA 325 mg plus DHA 175 mg twice daily for 3 months improved symptoms, particularly in participants with blepharitis or meibomian gland disease [119] (Table 1), while EPA 180 mg plus DHA 120 mg twice daily for 30 days reduced tear evaporation and improved tear breakup time and symptoms [128] (Table 1). In a 12-week trial involving 27 patients, fish oil providing EPA 1245 mg and DHA 540 mg daily improved ocular pain, tear breakup time, and rose bengal staining but not Schirmer I test results [127] (Table 1). Supplementation with 1.5 g/day omega-3 fatty acids for 12 weeks improved tear breakup time, Ocular Surface Disease Index scores, lid-margin inflammation, and meibomian-gland function [139] (Table 1). An 8-week high-DHA formulation also improved tear breakup time and MGD scores in patients with MGD-related DED [123] (Table 1).

Thus, positive findings from smaller or selected-population studies have not been confirmed in a large general DED population. Differences in disease subtype, baseline fatty-acid status, EPA/DHA dose and ratio, lipid form, comparator, and outcome selection may contribute to the heterogeneity.

##### Clinical and Observational Evidence in AMD

AREDS2 evaluated DHA/EPA as an addition to the background AREDS multi-nutrient formulation. The primary analysis did not demonstrate an additional reduction in progression to late AMD [140] (Table 1). A Cochrane review similarly concluded that omega-3 supplementation for up to 5 years did not reduce progression to late AMD or clinically meaningful visual-acuity loss [366].

Observational studies concern different exposures. A meta-analysis of prospective cohorts associated higher fish intake with lower AMD risk, with a more apparent association for tuna intake [367]. A 2024 cross-sectional analysis of National Health and Nutrition Examination Survey data also associated higher dietary DHA, docosapentaenoic acid, and EPA intake with lower odds of AMD, particularly early AMD [368]. These associations may reflect long-term dietary patterns and other lifestyle factors and do not outweigh the neutral supplementation evidence.

Overall, fish oil-derived EPA/DHA preparations have substantial structural, mechanistic, and preclinical support, but clinical efficacy remains uncertain. DREAM did not demonstrate superiority for DED, and AREDS2 did not demonstrate additional benefit for AMD progression. Evidence from isolated or concentrated EPA/DHA preparations and dietary fish intake should therefore remain distinct from evidence for source-defined whole fish oil.

#### 5.3.2. Krill Oil

Krill oil provides EPA and DHA largely in phospholipid-associated forms. In a 90-day randomised trial comparing krill oil, fish oil, and an olive oil placebo, tear osmolarity decreased in both marine oil groups, whereas improvement in Ocular Surface Disease Index scores was more apparent with krill oil [122] (Table 1). Current ocular evidence is limited to a small number of product-specific studies and does not establish a clinically meaningful advantage of krill oil over fish oil preparations.

#### 5.3.3. Algal Oils and Algal-Derived DHA Ingredients

Algal oils are produced from DHA- or EPA-accumulating microorganisms, including *Schizochytrium*, *Crypthecodinium*, *Nannochloropsis*, and *Phaeodactylum* species [369,370]. Depending on the strain and production process, the resulting oil may be predominantly rich in DHA or may provide appreciable amounts of EPA. These oils provide non-fish sources of long-chain omega-3 PUFAs and are used in nutritional supplements and infant formulas. Controlled cultivation can improve source traceability and reduce dependence on capture fisheries, although the fatty-acid profile, minor constituents, oxidative stability, and overall quality of the final product depend on the strain, culture conditions, extraction and purification procedures, and oxidation control [371,372,373]. Source-defined algal oils should therefore be distinguished from concentrated or purified algal-derived DHA or EPA ingredients.

The ocular relevance of algal oils and algal-derived DHA is concentrated primarily in early-life nutrition. DHA accumulates in the brain and retina during late fetal and early postnatal development and contributes to photoreceptor membrane organisation, synaptic function, and visual maturation [374,375]. Reviews support this physiological role but report heterogeneous effects of DHA supplementation on visual and neurodevelopmental outcomes. The observed effects vary according to the supplementation period, dose, baseline nutritional status, source of DHA, and outcome measures [376,377].

In lactating mothers, approximately 200 mg/day of high-DHA algal oil increased breast milk DHA and infant plasma-phospholipid DHA. Infant visual function did not differ at 4 or 8 months, although a higher Bayley Psychomotor Development Index was reported at 30 months [144] (Table 1). In the DIAMOND double-blind randomised trial, DHA-fortified infant formulas improved visual-evoked potential acuity at 12 months, but increasing the DHA concentration did not provide additional benefit [143] (Table 1). Another randomised trial comparing infant formulas containing DHA from DHASCO^® ^(DSM Nutritional Products, Columbia, MD, USA), with those containing DHA from the alternative algal single-cell oil source DHASCO^®^-B (DSM Nutritional Products, Columbia, MD, USA) showing comparable growth, tolerability, and erythrocyte DHA status through 120 days of age [145] (Table 1).

These studies concern high-DHA algal oil supplements or algal-derived DHA incorporated as an ingredient in multi-ingredient infant formulas. The findings support DHA delivery, nutritional adequacy, and selected aspects of early visual development, but they do not establish the efficacy of whole algal oil as an intervention for ocular disease. Outcomes from infant-formula studies should likewise be interpreted at the level of the complete formula unless the independent contribution of the algal-derived ingredient has been demonstrated.

Overall, algal oils and algal-derived DHA ingredients provide traceable non-fish sources of long-chain omega-3 PUFAs. Current human evidence is concentrated in maternal and infant nutrition, whereas direct clinical evidence for whole algal oil in adult DED, AMD, glaucoma, or other ocular diseases remains insufficient. Future studies should clearly identify whether the tested material is a source-defined whole algal oil, a concentrated DHA/EPA preparation, or one ingredient in a multi-ingredient product and should report the algal strain, fatty-acid composition, chemical form, dose, oxidative status, and clinically relevant ocular outcomes.

#### 5.3.4. Cod Liver Oil

Cod liver oil is a type of fish liver oil extracted primarily from the livers of cod species. Unlike conventional fish body oils, it contains preformed vitamins A and D in addition to EPA and DHA, although the concentrations of these nutrients vary substantially among products. Cod liver oil should therefore be classified as a multicomponent whole oil rather than as a preparation containing a single active constituent.

The potential ocular relevance of cod liver oil is based mainly on the established physiological roles of its individual nutrients. Vitamin A-derived retinaldehyde is essential for phototransduction and the retinal visual cycle. Vitamin A deficiency initially impairs rod function and causes night blindness and may subsequently result in reduced visual acuity, conjunctival and corneal xerosis, and keratomalacia [378]. DHA contributes to retinal membrane structure and photoreceptor function, whereas EPA and DHA serve as precursors of lipid mediators involved in inflammatory regulation and neuroprotective responses [379]. These constituent-level relationships provide biological plausibility but do not demonstrate that cod liver oil itself prevents or treats ocular disease.

Available clinical evidence is indirect. In retinal degenerative disease, a Cochrane review evaluated vitamin A, fish oil, and their combination for modifying the progression of retinitis pigmentosa. The review concluded that whether these interventions delay visual decline remains uncertain; these findings therefore cannot be interpreted as evidence that cod liver oil treats retinal degeneration [380]. In DED, short-term oral vitamin A supplementation has been reported to improve selected tear-film outcomes, but this study evaluated isolated vitamin A rather than cod liver oil as a whole oil intervention [381].

Safety considerations also differ from those for conventional fish-body oil because cod liver oil can provide substantial amounts of preformed vitamins A and D. Excessive intake may increase the risk of vitamin A or D toxicity, particularly when cod liver oil is consumed concurrently with other vitamin-containing supplements. Nutritional guidance has therefore recommended that the vitamin content of individual products and total intake from other supplements be considered [382].

Overall, cod liver oil has potential nutritional relevance as a multicomponent source of vitamins A and D and long-chain omega-3 PUFAs. However, current evidence more strongly supports the physiological and mechanistic roles of these individual nutrients than the disease-specific efficacy of cod liver oil as a whole. Direct clinical evidence that cod liver oil prevents or treats ocular disease remains insufficient. It should therefore be discussed as a potential nutritional source rather than as an established ophthalmic treatment.

## 6. Processing, Oxidative Stability, and Relevance to Ocular Applications

Oil processing can alter the identity, composition, oxidative status, contaminant burden, bioavailability, and formulation suitability of an intervention [383]. The principal stages include extraction, refining or concentration, and subsequent stabilisation. In this review, processing technologies are considered only where they materially affect constituents relevant to ocular health, product safety, or ophthalmic formulation performance.

In this review, compatibility with the whole natural oil category was determined by whether processing preserved a recognisable, source-derived, multicomponent lipid matrix rather than by whether the material was completely unprocessed. Mechanical pressing, aqueous or enzyme-assisted extraction, solvent extraction with adequate solvent removal, supercritical-CO_2_ extraction, and conventional refining or winterisation were considered compatible with this category when they did not selectively isolate individual constituents or substantially reconstruct the original lipid profile. Ultrasound-, microwave-, and pulsed-electric-field-assisted treatments were regarded as extraction-assistance technologies and did not themselves alter the intervention category. By contrast, substantial molecular concentration, selective purification, urea complexation, enzymatic re-esterification, microencapsulation, nanoemulsification, and nanoparticle construction were classified as producing concentrated, modified, or engineered lipid preparations rather than unmodified whole natural oils. When antioxidants or other ingredients were added, the product was interpreted at the formulation level if its biological or functional performance could not be attributed independently to the source oil.

### 6.1. Extraction and Retention of Oil Constituents

Mechanical pressing, solvent extraction, aqueous or enzymatic extraction, supercritical carbon dioxide extraction, and ultrasound-, microwave-, or pulsed-electric-field-assisted processes differ in extraction efficiency and retention of minor constituents [384]. Cold pressing limits thermal exposure and can improve retention of tocopherols, carotenoids, phytosterols, and phenolic compounds [385]. Hot pressing may increase oil recovery but can accelerate oxidation, reduce thermolabile constituents, and promote formation of heat-related contaminants when processing conditions are not adequately controlled [386]. Solvent extraction provides high oil recovery but requires desolventisation, refining, and control of residual solvents and processing-related compositional changes [387].

Aqueous and aqueous-enzymatic extraction use water and, where applicable, cell-wall- or protein-degrading enzymes to facilitate oil release [388]. These methods can reduce the risk of organic-solvent residues but may generate stable emulsions that complicate oil separation and influence the composition of the recovered oil [389,390].

Supercritical CO_2_ extraction permits selective recovery of lipophilic compounds at relatively low temperatures and is used for oils or fractions enriched in carotenoids, tocopherols, phytosterols, squalene, or EPA/DHA [391].

### 6.2. Refining, Fractionation, and Concentration

Refining removes phospholipids, free fatty acids, pigments, metals, volatile compounds, contaminants, and pre-existing oxidation products, thereby improving safety and storage stability [392]. Degumming, deacidification, bleaching, deodorisation, and winterisation can nevertheless reduce carotenoids, tocopherols, phytosterols, squalene, and other minor constituents. High-temperature or poorly controlled processing can also contribute to the formation of trans fatty acids, glycidyl esters, and 3-monochloropropane-1,2-diol esters [393,394,395]. The degree of refining is therefore relevant both to the retention of potentially beneficial constituents and to the removal or formation of compounds that may affect safety.

For fish and algal oils, molecular distillation, urea complexation, enzymatic re-esterification, low-temperature fractionation, or related procedures may be used to concentrate EPA/DHA and reduce cholesterol, free fatty acids, contaminants, or oxidation products [396].

### 6.3. Oxidative Stability, Storage, and Quality Reporting

Oils rich in EPA, DHA, α-linolenic acid, carotenoids, or other oxidation-sensitive constituents are vulnerable to oxidation during exposure to oxygen, light, heat, and pro-oxidant metals [73]. Oxidation can reduce nutrient content and generate primary and secondary oxidation products that alter biological activity, tolerability, and safety. This is particularly important when results from stored or poorly characterised oils are compared with those from freshly prepared or antioxidant-stabilised products.

Stability of whole oils can be improved through light- and oxygen-limiting containers, nitrogen flushing, low-temperature storage, and the addition of suitable antioxidants, whereas emulsification and microencapsulation produce formulation-level systems [397].

Studies of oils in ocular health should report the biological source and source tissue, extraction method, degree of refining or concentration, fatty-acid profile, chemical form, relevant minor constituents, oxidation indices, contaminant testing, storage conditions, and shelf life [398]. For topical ophthalmic products, sterility, pH, osmolarity, viscosity, particle-size distribution, preservative strategy, and ocular tolerability should also be documented [399]. These variables are necessary to determine whether differences among studies reflect the source oil, processing-related deterioration or enrichment, or the properties of the final formulation.

## 7. Routes of Exposure and Distinct Roles of Oils and Derived Lipids in Ophthalmology

Oils and derived lipids enter ophthalmic research through oral exposure, topical ocular–surface products, pharmaceutical oil phases, engineered delivery systems, and injectable or implantable formulations. These routes involve different materials and mechanisms: oral interventions alter systemic nutrient exposure, topical lipid products act primarily on the tear film and ocular surface, and pharmaceutical or engineered lipids mainly influence drug solubility, retention, penetration, and release. The evidence in this section is therefore organised according to route and the function of the material actually administered.

### 7.1. Oral Dietary and Supplement Exposure

#### 7.1.1. Oral Oils and Nutritional Supplements

Oral evidence includes source-defined whole oils consumed as foods or supplements, concentrated fatty-acid preparations, and multi-ingredient nutritional products [94,158,400]. These exposures should be recorded separately. For example, flaxseed oil is an ALA-rich whole oil, concentrated EPA/DHA products are constituent-level interventions, and infant formulas or AREDS-type products are multi-ingredient formulations.

Orally administered lipids undergo digestion, intestinal absorption, re-esterification, lipoprotein assembly, and systemic transport before influencing ocular tissues [401,402,403]. Their effects therefore depend on the administered dose and chemical form, gastrointestinal absorption, baseline nutritional status, tissue distribution, and concurrent dietary or pharmacological exposures [27,403,404]. The relevant composition may include EPA/DHA, ALA, γ-linolenic acid, carotenoids, tocopherols, phytosterols, or phenolic constituents, depending on the product [25,61,109].

Oral-intervention studies should report the biological source, extraction and refining status, quantified major fatty acids and relevant minor constituents, daily dose, chemical form, intervention duration, comparator, adherence, baseline diet, and concomitant supplementation [405,406]. Marine products should specify EPA and DHA content, ratio, and triacylglycerol, ethyl ester, phospholipid, or free fatty acid forms. ALA-rich plant oils require consideration of the limited conversion of ALA to long-chain omega-3 fatty acids [407]. Reporting minor constituents and oxidative status is also necessary when they form part of the proposed rationale [398].

Outcome selection should follow the ocular condition. DED studies commonly assess symptoms, tear breakup time, Schirmer testing, staining, tear osmolarity, lipid-layer thickness, and meibomian-gland function, whereas retinal studies may include macular pigment, optical coherence tomography, visual function, or electrophysiology. Dietary records, plasma or erythrocyte fatty-acid profiles, the omega-3 index, and circulating carotenoid or vitamin levels can support exposure assessment.

#### 7.1.2. Effects of Cooking on Oil Composition and Potential Ocular Relevance

The potential ocular consequences of cooking depend on both the retention of intact nutrients and the formation of lipid-oxidation products [73,408]. Oils consumed without heating or added after steaming or boiling are not directly exposed to prolonged thermal oxidation and are therefore more likely to retain tocopherols, phenolic compounds, carotenoids, and highly unsaturated fatty acids [409]. Short-duration sautéing with fresh oil produces a more complex balance between nutrient degradation and improved release from the food matrix [410]. Although heating may reduce some native antioxidants, disruption of plant tissues and transfer of carotenoids into the lipid phase can increase their extractability and facilitate incorporation into mixed micelles during digestion [411]. Cooking vegetables with extra-virgin olive oil increased the extractability of carotenoids and polyphenols, whereas the bioaccessibility of lutein and zeaxanthin from egg yolk differed among boiling, frying, and scrambling, with lower bioaccessibility reported for scrambled than for boiled eggs [412]. Moderate lipid-assisted cooking may therefore improve the bioaccessibility of macular carotenoids in selected food matrices, even when their absolute concentrations are partially reduced during heating [336]. These effects depend on the food matrix, oil type, temperature, and heating duration and should not be generalised to all oils or carotenoid-containing foods.

By contrast, prolonged pan-frying, deep-frying, and repeated reuse of cooking oil progressively deplete endogenous antioxidants and promote the accumulation of lipid hydroperoxides, aldehydes, polar compounds, and polymerised lipids [73,413,414]. These changes are particularly relevant to oils and foods rich in ALA, EPA, and DHA because increasing numbers of carbon–carbon double bonds increase susceptibility to non-enzymatic lipid oxidation [415]. In salmon, pan-frying and baking increased peroxide formation, while pan-frying additionally increased malondialdehyde and non-enzymatically oxidised products derived from omega-3 and omega-6 PUFAs [415].

From an ocular nutrition perspective, these findings are most relevant to macular-carotenoid bioaccessibility and the preservation of intact EPA and DHA [415]. The retina and RPE contain abundant oxidation-sensitive lipids, and local lipid peroxidation and oxidation-specific products are implicated in retinal oxidative injury and inflammatory activation [416]. Taken together, studies of thermally oxidised cooking oils and studies of retinal lipid peroxidation provide a biologically plausible but indirect link between repeated consumption of highly oxidised oils and retinal oxidative–inflammatory stress [414]. However, no direct clinical study was identified that compared specific cooking methods—such as steaming, boiling, sautéing, baking, or frying—in relation to the incidence or progression of AMD, DR, or other ocular diseases. Consequently, the available evidence supports method-dependent differences in nutrient retention, bioaccessibility, and lipid oxidation but does not establish that a particular cooking method prevents or causes a specific ocular disease.

### 7.2. Topical Ocular–Surface Applications

Topical products can increase local exposure but are limited by blinking, tear dilution, nasolacrimal drainage, and epithelial barriers [49]. Oils and derived lipids are used in lubricants, lipid-supplementing artificial tears, emulsions, ointments, and drug-containing formulations to improve tear-film lipid coverage, ocular–surface residence, or delivery of poorly soluble agents [131,132]. Such products require pharmaceutical-grade materials and formulation-specific ophthalmic evaluation [50,399,417].

#### 7.2.1. Whole Oil and Multi-Ingredient Lipid-Supplementing Products

Whole oils may be applied periocularly, whereas oils incorporated into multi-ingredient ocular–surface products should be interpreted at the formulation level. Emustil^®^ is discussed in Egg-Derived Phospholipids as Formulation Materials in Multi-Ingredient Ophthalmic Emulsions Section; the reported clinical and low-humidity findings concern the complete product [135,136] (Table 1).

#### 7.2.2. Pharmaceutical Oil Phases and Excipients

Castor oil is one of the most representative natural oil phases used in topical ophthalmic emulsions. The cyclosporine A ophthalmic emulsion Restasis^® ^(Allergan, Inc., Irvine, CA, USA) contains castor oil, glycerol, polysorbate 80, carbomer, purified water, and related excipients. In this formulation, castor oil mainly serves to dissolve highly lipophilic cyclosporine A and to form an oil-in-water emulsion that improves ocular-surface delivery [418]. Castor oil also serves as an oil phase in difluprednate ophthalmic emulsion [311] (Table 5).

Olive and sesame oils have also been used as non-aqueous solvents or oil-phase matrices for hydrophobic ophthalmic drugs [418]. Experimental fluocinolone–acetonide microemulsions containing pharmaceutical-grade cow ghee have shown enhanced ocular retention and penetration in animals [312] (Table 5).

Lanolin is an animal-derived wax rather than a triacylglycerol-rich dietary fat. Purified lanolin is used with petrolatum, mineral oil, or related materials as an ointment base and consistency-modifying excipient [51]. Its use may be limited by eyelid or periocular allergic contact dermatitis and therefore depends on purification, formulation quality, and individual sensitisation [419].

### 7.3. Engineered Lipid-Delivery Systems

#### 7.3.1. Oil-Based Matrices and Nanoemulsions

Oil-based ocular delivery systems use oils as structural matrices or internal phases to solubilise hydrophobic drugs and improve drug dispersion or ocular–surface contact [399,417,420]. A soybean oil oleogel/oleorod provided sustained epalrestat release and enhanced ocular retention without HET-CAM irritation [309], whereas selected almond-oil nanoemulsions showed suitable droplet-size distribution, surface charge, microviscosity, and physical stability [310]. Screening for a fingolimod nanoemulsion identified greater drug solubility in soybean and castor oils, which were subsequently selected for formulation development [313] (Table 5).

#### 7.3.2. Solid Lipid Nanoparticles (SLNs) and Nanostructured Lipid Carriers (NLCs)

SLNs use a predominantly solid lipid matrix, whereas NLCs combine solid lipids with liquid oils to increase drug loading and modify release [421,422]. Oil selection for NLCs depends on formulation-specific compatibility, oxidative stability, particle characteristics, and drug-solubilising capacity [423]. Mangiferin-loaded nanostructured lipid carriers showed acceptable compatibility in ARPE-19 cells and ex vivo models and retained antioxidant activity against hydrogen–peroxide-induced oxidative stress [314] (Table 5).

#### 7.3.3. Liposomes and Nanomicelles

Liposomes and nanomicelles use amphiphilic lipids or surfactants to organise the drug carrier. Liposomes consist of phospholipid bilayers and may incorporate soybean- or egg-derived phospholipids or lecithin as membrane-forming materials [424,425,426]. Nanomicelles, by contrast, self-assemble from amphiphilic materials to form a hydrophobic core capable of solubilising poorly water-soluble drugs [427,428]. In a phase III trial, the cyclosporine A nanomicellar product OTX-101 0.09% increased the proportion of participants achieving a 10 mm or greater improvement in Schirmer testing and improved corneal and conjunctival staining compared with vehicle [117] (Table 1). This clinical result concerns the engineered cyclosporine product and illustrates formulation-level efficacy.

### 7.4. Injectable, Implantable, and Long-Acting Systems

Injectable or implantable lipid systems are designed for sustained periocular or intraocular delivery in conditions such as glaucoma, uveitis, and retinal disease [429,430]. For intraocular injection or implantation, whole edible oils are generally not used directly as carrier materials. Lipid-based delivery systems intended for these routes are instead constructed from purified, pharmaceutical-grade lipids, such as phospholipids and cholesterol, or other well-characterised biomimetic materials.

Latanoprost-loaded liposomes prepared with egg phosphatidylcholine produced sustained drug release and prolonged intraocular-pressure reduction after subconjunctival injection in rabbits and remained stable for at least 6 months [315] (Table 5)**.** Related liposomal and vesicular systems have been investigated for anti-glaucoma, anti-inflammatory, anti-infective, and posterior-segment agents [431,432].

### 7.5. Route-Specific Safety and Quality Control

Safety requirements differ by route. Oral interventions require compositionally defined products, oxidation and contaminant control, dose monitoring, assessment of gastrointestinal adverse effects and drug interactions, and avoidance of excessive fat-soluble-vitamin exposure. Topical formulations additionally require sterility, appropriate pH and osmolarity, controlled viscosity and particle-size distribution, preservative compatibility, and evaluation of epithelial irritation, blurred vision, and sensitisation. Injectable, implantable, and long-acting systems require the most stringent control of sterility, endotoxins, aggregation, degradation products, tissue retention, inflammation, and long-term ocular toxicity [433].

For engineered carriers, surface charge, surfactants, droplet or particle size, and release behaviour can influence both retention and toxicity; excessive cationic charge or unsuitable surfactants may damage the corneal epithelium [420]. Clinical and formulation reports should therefore identify the role of each oil or lipid, characterise the final product, and distinguish tolerability of the carrier from safety and efficacy of the active drug.

## 8. Conclusions and Future Perspectives

Natural oils and fats are multicomponent lipid matrices composed predominantly of triacylglycerols with source-specific fatty-acid profiles, together with variable amounts of phospholipids, phytosterols, carotenoids, tocopherols, tocotrienols, phenolic compounds, and other minor lipophilic constituents. These components provide a biochemical basis for potential effects on ocular–surface homeostasis, tear-film stability, inflammatory and immune responses, lipid metabolism, mitochondrial function, and retinal cell integrity. Nevertheless, compositional complexity and biological plausibility should not be regarded as equivalent to clinical efficacy. The literature reviewed here encompasses whole oils and fats, isolated oil-associated constituents, multi-ingredient nutritional products, pharmaceutical oil phases, and engineered lipid-delivery systems. Findings from these categories are not interchangeable. Overall, current clinical evidence for whole natural oils and fats remains limited and varies across ocular conditions, with most findings supporting potential nutritional or adjunctive roles rather than established disease-specific therapeutic efficacy.

At the level of human evidence, the most direct findings are concentrated in DED and MGD, including studies of orally administered oils or fatty-acid supplements and product-specific studies of topical lipid-containing formulations. However, many of these studies involved relatively small samples, short intervention periods, or multi-ingredient products, making it difficult to determine the independent contribution of a particular oil. In addition, a large trial of concentrated EPA and DHA supplementation did not demonstrate superiority over placebo in an unselected DED population. For AMD and cataract, observational dietary associations cannot establish causality, while randomised trials of isolated nutrients or multi-ingredient supplements have not demonstrated a consistent overall additional clinical benefit attributable to their source oils. Evidence concerning uveitis, diabetic retinopathy, glaucoma, retinal degeneration, and most proposed retinal–neuroprotective effects currently relies largely on animal models, cell systems, or mechanistic inference. These findings provide important directions for further investigation but cannot yet be directly translated into clinical recommendations. Similarly, clinical findings from complete ophthalmic products should be interpreted at the formulation level rather than attributed independently to an individual oil or lipid component.

The interpretation of the available literature is further constrained by heterogeneity in the materials tested, study populations, doses, routes of administration, comparators, and outcome measures. Many studies provide insufficient information on oil source, fatty-acid composition, minor bioactive constituents, processing history, oxidative status, storage conditions, bioavailability, and long-term safety. Translation from experimental models is also uncertain because the doses, routes of exposure, and disease conditions used in animal and cell studies may not reflect clinically achievable ocular exposure. Consequently, evidence that an oil or oil-associated constituent regulates oxidative stress, inflammation, lipid homeostasis, mitochondrial function, barrier integrity, or neurodegenerative injury should be interpreted as mechanistic support rather than proof of disease-specific therapeutic efficacy.

Future research should first define and characterise the material actually tested. Future reviews and primary studies may benefit from more narrowly defined, disease- or intervention-specific objectives, which could permit more detailed and methodologically homogeneous assessment of efficacy, mechanisms, safety, and translational relevance. Whole oil interventions should be clearly distinguished from isolated fatty acids, carotenoids, polyphenols, multi-ingredient supplements, pharmaceutical excipients, and engineered lipid-delivery systems. Standardised reporting should include the biological source, extraction and refining methods, fatty-acid profile, concentrations of relevant minor constituents, acid and peroxide values, secondary oxidation products, contaminant residues, storage conditions, and stability throughout the study. Such characterisation is necessary to determine whether an observed effect is reproducible and whether it can reasonably be attributed to the tested oil, a particular constituent, or the complete formulation.

Mechanistic research should move beyond composition-based assumptions and examine dose–response relationships, tissue exposure, bioavailability, and disease-specific pathways. Lipidomics, metabolomics, transcriptomics, single-cell sequencing, and spatial omics may help identify molecular targets, tissue-specific responses, and interactions between local ocular tissues and systemic metabolic networks. However, these approaches should be linked to clinically relevant ocular endpoints. Effects on tear-film stability or epithelial repair cannot automatically be extrapolated to lens transparency, RPE homeostasis, retinal neuroprotection, or ocular-axis development. Age, metabolic status, dietary background, disease stage, concomitant treatment, and long-term safety should also be considered when identifying potentially responsive populations.

The ophthalmic application of natural oils used as oil phases and naturally derived pharmaceutical lipids also depends on dose, administration route, bioavailability, physicochemical stability, ocular tolerability, and safety. In formulation development, these materials may serve as oil phases, solvents, emulsifying materials, penetration-enhancing matrices, or components of nanoemulsions, liposomes, SLNs, NLCs, and other lipid-based delivery systems. Current formulation studies mainly support improvements in drug solubility, ocular–surface residence, permeability, stability, or controlled release. These outcomes demonstrate delivery performance but do not necessarily indicate an intrinsic therapeutic effect of the carrier oil. Future studies should therefore compare complete formulations with appropriate oil-free, vehicle, and active-drug controls and should separately evaluate physicochemical performance, ocular pharmacokinetics, tolerability, and therapeutic efficacy.

Ultimately, adequately powered randomised controlled trials using well-characterised interventions, appropriate comparators, clinically relevant endpoints, and sufficiently long follow-up are required to clarify efficacy, effective dose, safety, and target populations. Until more robust evidence becomes available, natural oils and fats are most appropriately considered potential nutritional modulators, adjunctive ocular–surface lipid-replenishing interventions, or supportive interventions. Oil-containing ophthalmic formulations should likewise be interpreted according to the performance and clinical outcomes of the complete product unless the independent contribution of an individual oil has been demonstrated. This evidence-based distinction will help define where particular oils, oil-associated constituents, or lipid-containing formulations may have clinically meaningful roles in ocular-health support and ophthalmic drug delivery.

## Data Availability

No new data were created or analyzed in this study. Data sharing is not applicable to this article.

## Abbreviations

ABCA4	ATP-binding cassette subfamily A member 4
ALA	α-linolenic acid
AMD	Age-related macular degeneration
ARA	Arachidonic acid
AREDS	Age-Related Eye Disease Study
AREDS2	Age-Related Eye Disease Study 2
CoQ10	Coenzyme Q10
DED	Dry eye disease
DEQ-5	5-item Dry Eye Questionnaire
DHA	Docosahexaenoic acid
DHASCO^®^	DHA single-cell oil
DPA	Docosapentaenoic acid
DR	Diabetic retinopathy
EggPC	Egg phosphatidylcholine
EPA	Eicosapentaenoic acid
EPO	Evening primrose oil
EVOO	Extra virgin olive oil
GLA	γ-linolenic acid
GSH	Glutathione
HIF-1α	Hypoxia-inducible factor-1α
IL-1β	Interleukin-1 beta
IL-6	Interleukin-6
LA	Linoleic acid
MGD	Meibomian gland dysfunction
MNU	N-methyl-N-nitrosourea
MUFAs	Monounsaturated fatty acids
NF-κB	Nuclear factor kappa B
NIBUT	Non-invasive tear breakup time
NLCs	Nanostructured lipid carriers
NLRP3	NOD-like receptor family pyrin domain containing 3
NPD1	Neuroprotectin D1
OCT	Optical coherence tomography
OSDI	Ocular Surface Disease Index
PEF	Pulsed electric field
PUFAs	Polyunsaturated fatty acids
RNS	Reactive nitrogen species
ROS	Reactive oxygen species
RPE	Retinal pigment epithelium
SBO	Sea buckthorn oil
SFAs	Saturated fatty acids
SFE-CO_2_	Supercritical CO_2_ extraction
SLNs	Solid lipid nanoparticles
TAGs	Triacylglycerols
TBUT	Tear breakup time
TNF-α	Tumour necrosis factor-α
VCO	Virgin coconut oil
VEGF	Vascular endothelial growth factor
